# A systematic review and meta-analysis on utilizing anti-CD19 chimeric antigen receptor T-cell therapy as a second-line treatment for relapsed and refractory diffuse large B-cell lymphoma

**DOI:** 10.3389/fonc.2024.1407001

**Published:** 2024-07-18

**Authors:** Kanwal Asghar, Maryam Zafar, Eva Holland, Ali Bin Abduljabbar, Sara A. Albagoush, Noureen Asghar, Akshat Sood, Jalal M. Dufani, Joseph Thirumalaredy, Bradley DeVrieze, Abubakar Tauseef, Muhammad Husnain

**Affiliations:** ^1^ Department of Medicine, Dow Medical College, Karachi, Pakistan; ^2^ School of Medicine, Creighton University, Omaha, NE, United States; ^3^ Department of Internal Medicine, Creighton University, Omaha, NE, United States; ^4^ Department of Medicine, University of Arizona, Tucson, AZ, United States

**Keywords:** DLBCL - diffuse large B cell lymphoma, CAR-T cell therapy, relapsed and refractory, second line treatment, standard of care

## Abstract

**Introduction:**

Inconsistent results observed in recent phase III trials assessing chimeric antigenic receptor T (CAR-T) cell therapy as a second-line treatment compared to standard of care (SOC) in patients with relapsed/refractory diffuse large B-cell lymphoma (R/R DLBCL) prompted a meta-analysis to assess the effectiveness of CAR-T cell therapy in this setting.

**Methods:**

Random-effects meta-analysis was conducted to pool effect estimates for comparison between CAR-T cell therapy and SOC. Mixed treatment comparisons were made using a frequentist network meta-analysis approach.

**Results:**

Meta-analysis of three trials with 865 patients showed significant improvement in event-free survival (EFS: HR: 0.51; 95% CI: 0.27-0.97; I2: 92%), progression-free survival (PFS: HR: 0.47; 95% CI: 0.37-0.60; I2: 0%) with CAR-T cell therapy compared to SOC. Although there was a signal of potential overall survival (OS) improvement with CAR-T cell therapy, the difference was not statistically significant between the two groups (HR 0.76; 95% CI: 0.56 to 1.03; I2: 29%). Mixed treatment comparisons showed significant EFS benefit with liso-cel (HR: 0.37; 95% CI: 0.22-0.61) and axi-cel (HR: 0.42; 95% CI: 0.29-0.61) compared to tisa-cel.

**Discussion:**

CAR-T cell therapy, as a second-line treatment, appears to be effective in achieving higher response rates and delaying the disease progression compared to SOC in R/R DLBCL.

## Introduction

Diffuse large B cell lymphoma (DLBCL), an aggressive subtype of non-Hodgkin lymphoma, is a curable disease, with long term remissions seen in 60-70% of patients treated with the standard first-line rituximab-based chemoimmunotherapy (CIT) (1). For patients with refractory and/or relapsed disease, the standard of care (SOC) consists of high dose chemotherapy followed by autologous stem cell transplantation (ASCT) in patients with chemosensitive disease. Nearly half of these patients can achieve long term remission with ASCT (2). In contrast, patients who are refractory to first line treatment or relapse shortly after, have dismal outcomes with median overall survival of 6 months (1). In current practice, patients who do not respond to salvage chemotherapy (therefore unable to proceed to ASCT) or relapse after ASCT can be treated with an approved anti CD-19 chimeric antigen receptor T cell (CAR-T) therapy such as axicabtagene ciloleucel (axi-cel), lisocabtangene maraleucel (liso-cel), and tisagenlecleucel (tisa-cel) in third line setting or later (3–5).

Recently, three randomized controlled trials (RCTs) TRANSFORM (6), ZUMA-7 (7), and BELINDA (8) were conducted in hopes to establish CAR-T as the second line of treatment in DLBCL. These trials compared the outcomes of anti-CD19 CAR-T cell therapy against SOC in patients with either primary refractory DLBCL or relapsing within 12 months after first line CIT. TRANSFORM (6) and ZUMA-7 (7) reported positive outcomes of CAR-T cell therapy with respect to event free survival compared to SOC while in BELINDA trial (8), CAR-T cell therapy failed to improve event free survival compared to SOC. Thus, we performed a meta-analysis to quantify the relative and absolute benefit of CAR-T cell therapy compared to SOC as second line treatment for R/R DLBCL.

## Methods

**Figure 1:**
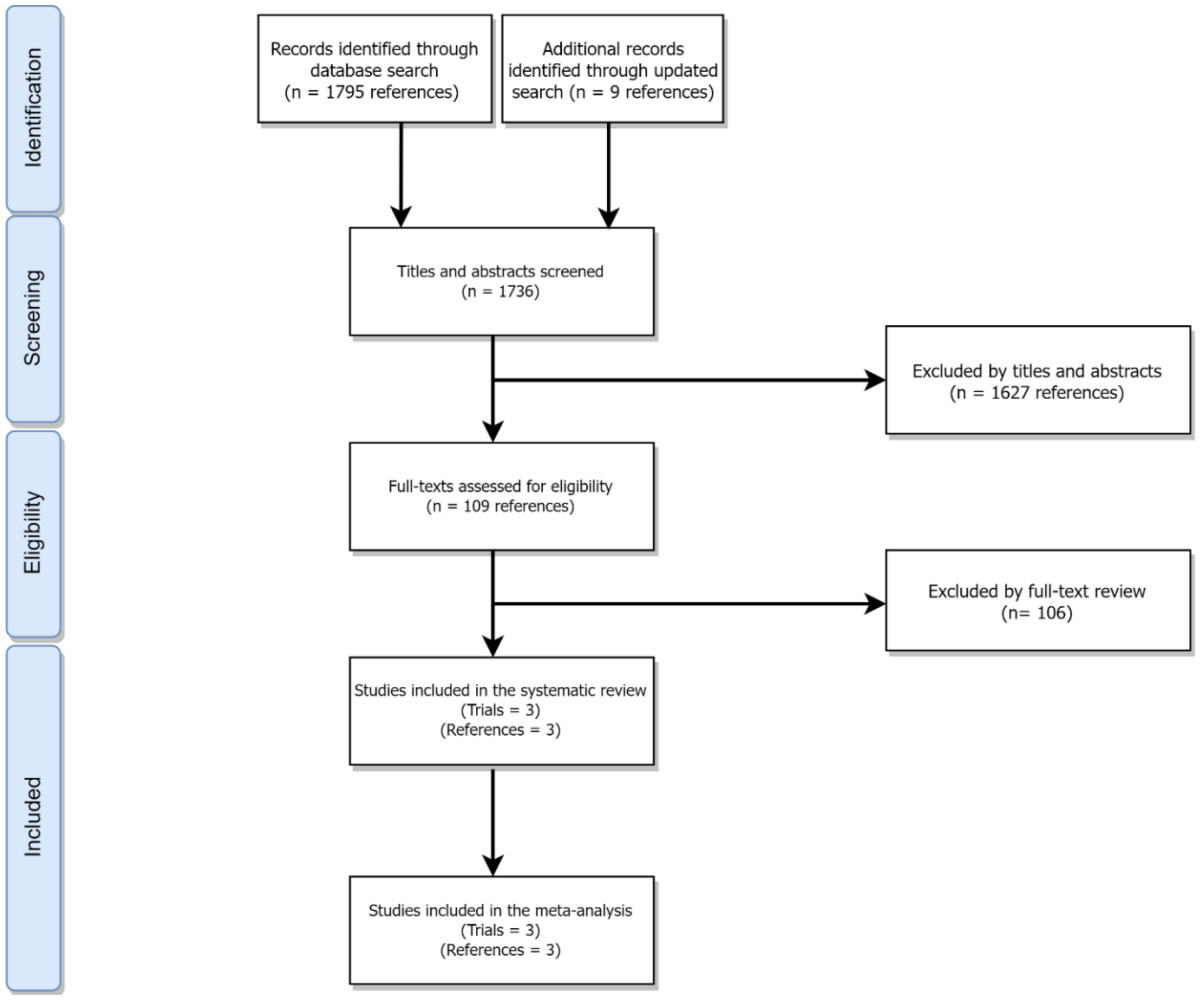
PRISMA flowchart outlining the process of study selection.

This systematic review and meta-analysis is reported in accordance with the Preferred Reporting Items for Systematic Review and Meta-Analyses (PRISMA) statement (9) (**Figure 1** and **eMethods 1efd**).

### Search strategy and selection criteria

Using the Ovid interface, MEDLINE(R) and Epub Ahead of Print, In-Process & Other Non-Indexed Citations, and Daily; EMBASE; Cochrane Central Register of Controlled Trials; Cochrane Database of Systematic Reviews were searched from each database inception through February 11^th^, 2022 to identify full-text or abstract publications of phase III randomized controlled trials (RCTs) assessing the effectiveness of CAR-T cell therapy in patients with previously treated DLBCL as compared to standard of care (SOC) (**eMethods 2 in the Supplementary**). Additionally, an updated search was conducted on July 1^st^ through Google Scholar to identify new trials. Non-randomized clinical trials, phase I/II/IV and observational studies, and articles in non-English language were excluded. Two independent reviewers (MZ and NA) screened relevant trials. Any discrepancy between the two reviewers were resolved by consensus and with input from a third reviewer (MH).

### Data extraction and quality assessment

Data was then extracted from the trials deemed eligible for inclusion using a pre-defined structured data collection instrument. The extracted data included (but was not limited to) baseline trial characteristics (study identification information, year of publication, trial design, number of arms, type of CAR-T cell product, and primary endpoint), population characteristics (age, total number of participants in each arm, histologic types, and disease status at entry), and outcome results. Two reviewers (MZ and NA) independently carried out the process of data extraction and subsequently assessed risk of bias in these studies using Cochrane Risk of Bias tool version 2 (10). Any disagreement between the reviewers were resolved by consensus and with input from a third reviewer (MH).

### Outcomes of interest

Patient important efficacy endpoints included event-free survival (EFS), progression-free survival (PFS), overall survival (OS), objective response rate (ORR) including complete- and partial- response (CR and PR) while any adverse events, cytokine release syndrome (CRS) and neurological toxicity (NT) was assessed as safety outcomes.

### Statistical analysis

#### Pairwise meta-analysis

A DerSimonian-Laird random-effects meta-analysis was used. Precomputed hazard ratios (HR) with their corresponding 95% confidence intervals (95% CI) were pooled using an inverse-variance weighted approach after logarithmic transformation. Raw binary data were pooled using the Mantel-Haenszel weighted approach; treatment effects were expressed as relative risks (RRs) with associated 95% confidence intervals (CI). Trial level incidence rates for CAR-T specific safety outcomes (CRS and NT) were computed, and subsequently meta-analyzed using the Freeman-Tukey transformation method to estimate incidence of events. Clopper-Pearson method was used to estimate the associated 95% CI. Cochran’s Q statistical test was used to assess the presence of statistically significant variance not explained by chance, while I^2^ statistical test was used to quantify the total observed variability, due to between-study heterogeneity. I^2^ values >75% indicated substantial heterogeneity.

Since most patients in the SOC arm in the included trials (6–8) did not respond to salvage chemotherapy and were not able to proceed to ASCT, we conducted a posthoc analysis to explore complete response rates in patients who received CAR-T cell therapy compared to those who managed to undergo ASCT.

Pre-specified subgroup analyses were conducted by age (<65 and >65 years), non-Hodgkin lymphoma (NHL) subtype, DLBCL molecular subtype (cell of origin), and prior response status. These analyses were subject to availability of data. A P-value of <0.1 indicated statistically significant effect modification.

#### Mixed treatment comparisons

Mixed treatment comparisons were made using a network meta-analysis within the frequentist framework to assess comparative effectiveness of different CAR-T cell products; the choice of the meta-analytic model was made based on sparsity of direct evidence and geometric structure of the network; fixed-effect model was used if the direct evidence was sparse with open network as the assessment of between study heterogeneity is not reliable in such networks (11). Relative treatment rankings were evaluated using P-score and were assessed in congruency with pairwise estimates. Higher ranking indicated better effectiveness of a treatment. Mixed treatment comparisons for each outcome of interest were presented as a color-coded league table. All statistical analyses were conducted in R project for statistical computing (version 4.1.1).

### Certainty of evidence

Certainty of evidence for direct comparisons between CAR-T cell therapy and SOC was assessed using Grading of Recommendations Assessment, Development, and Evaluation (GRADE) approach (12). The effect estimates for each outcome were carefully examined for risk of bias, inconsistency, indirectness, imprecision, and publication bias. Corresponding risks with CAR-T cell therapy were estimated using the assumed baseline risk of an event with SOC (as abstracted from included trials) and relative effect estimates (from the results of this meta-analysis). Absolute risk difference was then calculated as the difference between the corresponding intervention risk and assumed risk with SOC.

## Results

Of 1803 studies initially identified (**Figure 1**), three trials (6–8) with a total of 865 patients and assessing axi-cel, tisa-cel, liso-cel were included in this systematic review and meta-analysis (**Table 1**). The overall risk of bias for all studies was low (**eFigure 1 in the Supplementary**).

**Table 1:** Description of trial characteristics and outcomes.

	ZUMA 7		TRANSFORM		BELINDA	
	Axi-cel group	SOC group	Liso-cel	SOC group	Tisa-cel	SOC group
Patient Characteristics						
Total patients (N)	180	179	92	92	162	160
Median age (range) — year	58 (21–80)	60 (26–81)	60 (53.5-67.5)	58 (42-65)	59.5 (19–79)	58 (19–77)
Age ≥65 year — no. (%)	51 (28)	58 (32)	36 (39)	25 (27)	54 (33)	46 (29)
Male — no. (%)	110 (61)	127 (71)	44 (48)	61 (66)	103 (64)	98 (61)
Disease stage — no. (%)						
I or II	41 (23)	33 (18)	24 (26)	29 (31)	55 (34)	62 (39)
III or IV	139 (77)	146 (82)	68 (74)	63 (68)	107 (66)	98 (61)
Histological type — no. (%)						
DLBCL, NOS	126 ( 70)	120 (67)	53 (58)	49 (53)	101 (62)	112 (70)
HGBL, DH	31 (17)	25(14)	22 (24)	21 (23)	32 (20)	19 (12)
HGBL, NOS	0	1 (1)	NR	NR	7 (4)	8 (5)
FL grade 3B	0	0	1 (1)	0	5 (3)	1 (1)
PMBL	0	0	8 (9)	10 (11)	12 (7)	13 (8)
Other or missing	23 (13)	33 (18)	8 (9)	12 (13)	5 (3)	7 (4)
Molecular Subgroup — no. (%)						
Germinal center B-cell–like	109 (61)	99 (55)	45 (49)	40 (43)	46 (28)	63 (39)
Activated B-cell–like	16 (9)	9 (5)	21 (23)	29 (32)	52 (32)	42 (26)
Not applicable	10 (6)	16 (9)	NR	NR	NR	NR
Unclassified	17 (9)	14 (8)	25 (27)	23 (25)	3 (2)	7 (4)
Missing data	28 (16)	41 (23)	1 (1)	0		
Second-line age-adjusted IPI of 2 or 3 — no. (%)	82 (46)	79 (44)	36 (39)	37 (40)	NR	NR
IPI score > 2 — no. (%)	NR	NR	NR	NR	106 (65)	92 (58)
Disease status at study entry^a^ — no. (%)						
Refractory to any therapy	133 (74)	131 (73)	67 (73)	68 (74)	107 (66)	107 (67)
Relapsed	47 (26)	48 (27)	25 (27)	24 (26)	55 (34)	53 (33)
Inclusion criteria	Refractory or relapsed within 12 months of 1st line		Refractory or relapsed within 12 months of 1st line		Refractory or relapsed within 12 months of 1st line	
CAR-T Therapy						
CAR-T product	Axi-cel		Liso-cel		Tisa-cel	
CAR-T target	CD19		CD19		CD19	
Costimulation	CD28/CD3zeta		4-1BB/CD3zeta		4-1BB/CD3zeta	
Vector	Gamma retrovirus		Lentivirus		Lentivirus	
T cell selection	No		Yes		Yes	
CD4:CD8 selection	No		CD4:CD8 infused in a 1:1 ratio		No	
CAR T-cell dose	2×10^6^ cells/kg		1 × 10^8^ cells		0.6–6 ×10^8^ cells / Median, 2.9× 10^8^ cells	
CAR-T infused — no. (%)	170 (94)		90 (98)		155 (96)	
Median time from randomization to CAR T-cell infusion — days	29		34		NR	
Median time from leukapheresis to CAR T-cell release — days	13		36		52 23.5 (U.S.); 28 (non-U.S. countries)	
Lymphodepletion	Flu 30 mg/m^2^ × 3 day; Cy 500 mg/m2 × 3 days		Flu 30 mg/m^2^ × 3 day; Cy 300 mg/m2 × 3 days		Flu 25 mg/m^2^ × 3 day; Cy 250 mg/m2 ×3 days	
							Bridging regimen	Steroids only- (no chemotherapy)		Protocol defined SOC regimen to stabiles their disease during Liso-cell manufacturing		Chemotherapy optional 1 cycle = 36%; 2+ cycles = 47%	
Received — no. (%)	65 (36)		58 (63)		135 (83%)	
Salvage regimen	2nd line CIT		2nd line CIT		2nd line CIT; 3rd line	
ASCT — no. (%)	64 (36)		42(46)		52 (32)	
Crossover to CAR-T — no. (%)	100 (56)		47 (55)		81 (51)	
Primary end point	EFS		EFS per IRC		EFS after 12 W	
ORR — no. (%)	150 (83)	90 (50)	79 (86)	44 (48)	75 (46)	68 (43)
CR — no. (%)	117 (65)	58 (32)	61 (66)	36 (39)	46 (28)	44 (28)
EFS median (months)	8.3	2	10.1	2.3	3	3
OS median (months)	NR	35.1	NR	16.4	16.9	15.3
Median follow up	24.9	24.9	6.2	6.2	10	

The risk in the intervention group (and its 95% confidence interval) is based on the assumed risk of an event in the comparator group (as abstracted from included trials) and the relative effect of the intervention (and its 95% CI). High certainty: we are very confident that the true effect lies close to that of the estimate of the effect. Moderate certainty: we are moderately confident in the effect estimate: the true effect is likely to be close to the estimate of the effect, but there is a possibility that it is substantially different. Low certainty: our confidence in the effect estimate is limited: the true effect may be substantially different from the estimate of the effect. Very low certainty: we have very little confidence in the effect estimate: the true effect is likely to be substantially different from the estimate of effect. CI: confidence interval; HR: hazard ratio; RR: risk ratio.

^a^Rated down one level due to serious inconsistency due to statistically significant heterogeneity in treatment effects as well as imprecision due to the small overall sample size.

^b^Rated down 2 levels for very serious imprecision due to wide confidence intervals and treatment effects indicating both substantial potential benefit and harm, as well as the small sample size and number of events.

^c^Rated down one level due to imprecision that relates to overall small sample size.

### Pairwise meta-analysis

A total of 260 EFS events (59.9%) were observed with CAR-T cell therapy as compared to 311 EFS events (72.5%) observed with SOC. The difference was statistically significant (HR: 0.51; 95% CI: 0.27-0.97; I^2^: 92%). In terms of PFS, a total of 125 events (45.9%) were observed with CAR-T cell therapy as compared to 174 events observed with SOC. The difference was statistically significant (HR: 0.47; 95% CI: 0.37-0.60; I^2^: 0%). Although only 137 deaths (31.5%) were observed with CAR-T cell therapy compared to 150 deaths (34.8%) with SOC, the difference was not statistically significant (HR: 0.76; 95% CI: 0.56-1.03; I^2^: 29%). These results are shown in **eFigures 2**-**4 in the Supplementary**. Similarly, while patients on CAR-T cell therapy were more likely to achieve an objective response (RR: 1.49; 95% CI: 1.13-1.97; I^2^: 81%; **eFigure 5 in the Supplementary**) and CR (RR: 1.55; 95% CI: 1.07-2.24; I^2^: 79%; **eFigure 6 in the Supplementary**), PR was not different between CAR-T cell therapy and SOC (RR: 1.26; 95% CI: 0.86-1.85, I^2^: 33%; **eFigure 7 in the Supplementary**). Lower CR rates with CAR-T cell therapy were observed when the analysis was limited to a comparison between patients who responded to salvage chemotherapy and underwent ASCT (RR: 0.61; 95% CI: 0.44-0.85; I^2^: 88%; **eFigure 8 in the Supplementary**).

The safety profile of CAR-T cell therapy relative to SOC showed no statistically significant difference for all grade and grade ≥3 any AE (RR: 1.01; 95% CI: 0.98-1.05; I^2^: 82%, RR: 1.05; 95% CI: 0.93-1.18; I^2^: 82%, respectively) as shown in **eFigures 9**, **10 in the Supplementary**. The incidence rate of all-grade CRS was 69.8% (95% CI: 39.5-92.9; I^2^: 97%) and for grade ≥3 CRS was 4.19% (95% CI: 1.60-7.80; I^2^: 57%) as shown in **eFigures 11**, **12 in the Supplementary**. Consistent results were observed for all grade NT with an incidence rate of 25.0% (95% CI: 1.87-61.7; I^2^: 98%) and for grade ≥3 NT, 7.57% (95% CI: 0.20-22.6; I^2^: 95%) (**eFigures 13**, **14 in the Supplementary**).

EFS benefit was consistent across prespecified subgroups and no statistically significant effect modification was observed as shown **in eFigures 15**-**18 in the Supplementary**.

### Mixed treatment comparisons

A detailed geometrical representation of network is shown in **eFigure 19 in the Supplementary**. Results from the fixed effect model are reported here considering the open network geometry which had sparse direct evidence. Mixed treatment comparisons were also made using random-effects model (not reported here) which indicated consistent direction of the results but wider confidence intervals.

**Figure 2:**
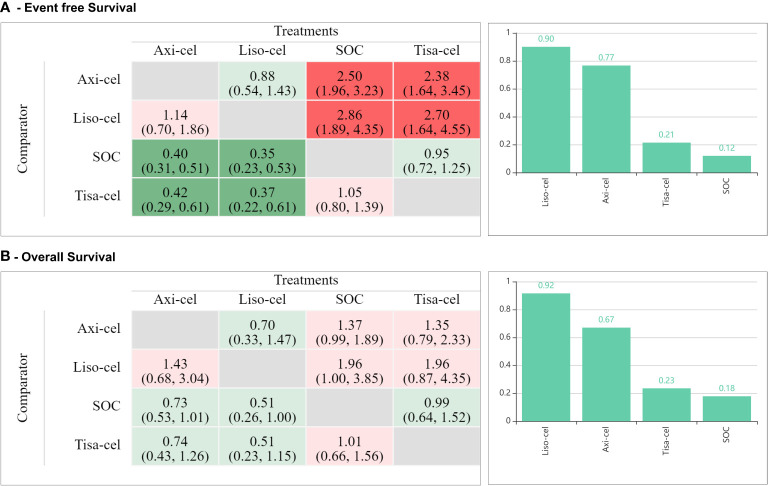
Mixed treatment comparisons for **(A)** event-free survival, and **(B)** overall survival.

**Figure 3:**
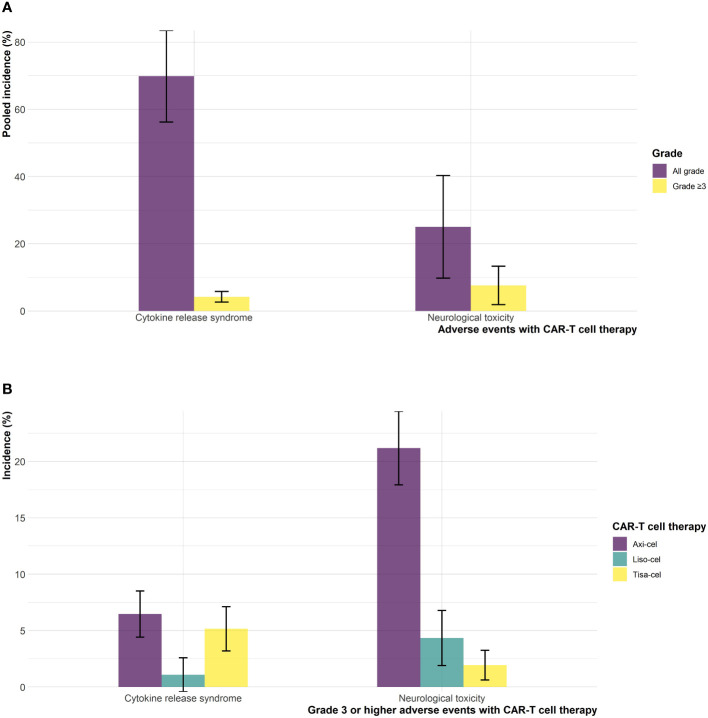
Cytokine release syndrome and neurological toxicities **(A)** pooled incidence, and **(B)** incidence across different CAR-T cell products.

Mixed treatment comparisons showed significant EFS benefit with axi-cel (HR: 0.42; 95% CI: 0.29-0.61) and liso-cel (HR: 0.37; 95% CI: 0.22-0.61) compared to tisa-cel. No significant difference was observed between axi-cel and liso-cel (HR: 1.14; 95% CI: 0.70-1.86) with regards to EFS outcome as shown in **Figure 2A**. In terms of OS, no significant differences were observed among different CAR-T cell products. (axi-cel vs. tisa-cel - HR: 0.74; 95% CI: 0.43-1.26, liso-cel vs. tisa cel – HR: 0.51; 95% CI: 0.23-1.15, axi-cel vs. liso-cel – HR: 1.43; 95% CI: 0.68-3.04; **Figure 2B**). Objective response rates were 83.3%, 85.8%, and 46.2% in patients who received axi-cel, liso-cel, and tisa-cel, respectively. Objective response was significantly more likely with axi-cel (83.3%; RR: 1.52; 95% CI: 1.14-2.04), and liso-cel (85.8%; RR: 1.65; 95% CI: 1.18-2.30) when compared to tisa-cel. Similar results were observed for CR with 65% of the patients achieving CR with axi-cel, 66.3% with liso-cel and only 28.3% with tisa-cel. Complete response was significantly more likely to occur with axi-cel (RR: 1.94; 95% CI: 1.27-2.97), and liso-cel (RR: 1.64; 95% CI: 1.04-2.59) when compared to tisa-cel as shown in **eFigures 20**, **21 in the Supplementary**. In terms of grade ≥3 any AE, the safety profiles of different CAR-T products were different and tisa-cel was observed to be the safest among other options (axi-cel vs. tisa-cel - RR: 2.55; 95% CI: 2.06-3.14, liso-cel vs. tisa cel – RR: 1.72; 95% CI: 1.45-2.05; **eFigure 22 in the Supplementary**). The results of CAR-T cell specific toxicity are shown in **Figure 3**.

## Discussion

The results of this systematic review and meta-analysis showed that treatment of DLBCL with CAR-T cell therapy in second line setting achieves significantly higher response rates, longer duration of remission, and delayed disease progression with no statistically significant increase in overall toxicity as compared to SOC. Although, there appears to be a signal of overall survival benefit with CAR-T cell therapy, the difference was not statistically significant at current follow up (**Table 2**). Treatment related mortality was comparable with 4% (18/434) in the CAR-T cell therapy arm versus 3.9% (17/431) in the SOC arm. The incidence rates for all-grade and grade ≥3 CRS were approximately 70%, and 4%, respectively in patients who received CAR-T cell therapy. All-grade and grade ≥3 NT were observed in 25% and 7.5% of the patients who received CAR-T cell therapy. Among different CAR-T cell products, delayed disease progression, and increased objective response were observed with axi-cel and liso-cel as compared to tisa-cel. In terms of OS improvement, no difference was observed among different CAR-T cell products at current follow-up. However, the relative safety of different CAR-T cell products was different, with tisa-cel being the safest among others.

**Table 2:** Evidence profile.

Outcomes	Number of participants	Relative effect	Anticipated absolute effects (95% CI)		Certainty of the evidence
	(studies)	(95% CI)	Risk with SOC	Risk difference with CAR-T	(GRADE)
**Event-free survival**	865	**HR 0.51**	720 per 1,000	**242 fewer per 1,000**	Moderate^a^
	(3 RCTs)	(0.27 to 0.97)		(from 429 fewer to 11 fewer)	
**Overall survival**	865	**HR 0.76**	348 per 1,000	**70 fewer per 1,000**	Low^b^
	(3 RCTs)	(0.56 to 1.03)		(from 135 fewer to 8 more)	
**Progression-free survival**	543	**HR 0.47**	642 per 1,000	**259 fewer per 1,000**	Moderate^c^
	(2 RCTs)	(0.37 to 0.60)		(from 326 fewer to 182 fewer)	
**Objective response rate**	865	**RR 1.49**	701 per 1,000	**343 more per 1,000**	Moderate^a^
	(3 RCTs)	(1.13 to 1.97)		(from 91 more to 680 more)	
**Complete response**	865	**RR 1.55**	320 per 1,000	**176 more per 1,000**	Moderate^a^
	(3 RCTs)	(1.07 to 2.24)		(from 22 more to 397 more)	
**Partial response**	865	**RR 1.26**	148 per 1,000	**39 more per 1,000**	Low^b^
	(3 RCTs)	(0.86 to 1.85)		(from 21 fewer to 126 more)	
**All grade any adverse event**	843	**RR 1.01**	981 per 1,000	**10 more per 1,000**	Moderate^c^
	(3 RCTs)	(0.98 to 1.05)		(from 20 fewer to 49 more)	
**Grade 3 or higher any adverse event**	843	**RR 1.05**	866 per 1,000	**43 more per 1,000**	Moderate^c^
	(3 RCTs)	(0.93 to 1.18)		(from 61 fewer to 156 more)	
**High certainty benefit**	**Moderate certainty benefit**	**Low certainty benefit**	**Low certainty harm**	**Moderate certainty harm**	**High certainty harm**

The risk in the intervention group (and its 95% confidence interval) is based on the assumed risk of an event in the comparator group (as abstracted from included trials) and the relative effect of the intervention (and its 95% CI). High certainty: we are very confident that the true effect lies close to that of the estimate of the effect. Moderate certainty: we are moderately confident in the effect estimate: the true effect is likely to be close to the estimate of the effect, but there is a possibility that it is substantially different. Low certainty: our confidence in the effect estimate is limited: the true effect may be substantially different from the estimate of the effect. Very low certainty: we have very little confidence in the effect estimate: the true effect is likely to be substantially different from the estimate of effect. CI: confidence interval; HR: hazard ratio; RR: risk ratio.

^a^Rated down one level due to serious inconsistency due to statistically significant heterogeneity in treatment effects as well as imprecision due to the small overall sample size.

^b^Rated down 2 levels for very serious imprecision due to wide confidence intervals and treatment effects indicating both substantial potential benefit and harm, as well as the small sample size and number of events.

^c^Rated down one level due to imprecision that relates to overall small sample size.

The results of this study suggests statistically significant improvement in EFS with liso-cel (6) and axi-cel (7) as compared to tisa-cel (8). However, it is important to view these results in the context of the current evidence and the differences across the included trials. First, there was variability in EFS definitions; more specifically, the BELINDA trial defined EFS as the time from randomization to either progressive or stable disease at or after 12 weeks in addition to death at any time. This suggests that the events occurring prior to week 12 would be counted as events in the ZUMA-7 (7) and TRANSFORM (6) trials but not in the BELINDA trial (8) which could explain this underlying heterogeneity and discrepant EFS observed across these trials (**eTable 1 in the Supplementary**). Considering these differences in EFS across trials, PFS might have been a more optimal endpoint, however, PFS was not reported in the BELINDA trial (8). Second, longer median time from leukapheresis to CAR-T cell infusion (52 days in BELINDA (8)) compared to a median time from randomization to infusion of 29- and 34-days in the ZUMA-7 (7), and TRANSFORM (6) trials, respectively might explain the disparate outcomes observed in these trials. Third, mixed treatment comparisons showed that the response rates were significantly lower with tisa-cel as compared to axi-cel and liso-cel. This pattern is consistent with the findings from studies assessing CAR-T cell therapy in the third line setting (58% CR with axi-cel from the ZUMA-1 trial (3), 53% CR with liso-cel from the TRANSCEND trial (5); and 40% CR with tisa-cel from the JULIET trial (4)). Interestingly, the complete response to tisa-cel in the second line setting was observed in only 28.3% of the patients in the BELINDA trial which is even lower than the 40% observed in the JUILET trial (4) in the third line setting. This lower complete response rate with tisa-cel in the second line setting may potentially explain the negative EFS outcome. Fourth, it is also important to highlight that trials were different in their use of bridging therapies prior to CAR-T cell therapy. ZUMA-7 (7) only allowed the use of glucocorticoids and did not allow the use of bridging chemotherapy prior to CAR-T cell therapy which by design excludes patients with highly aggressive and advanced disease; conversely, approximately 60% of the patients in TRANSFORM trial (6) and 83% in BELINDA trial (8) received bridging chemotherapy. However, patients in the trial TRANSFORM trial were only allowed one cycle of bridging chemotherapy while 47% patients in the BELINDA trial received (3) 2 cycles. Patients who receive bridging chemotherapy are known to harbor worse prognosis than those who do not, and these patients are more reflective of the real-world clinical setting (13). Fifth, while BELINDA (8) and TRANSFORM (6) trials allowed the receipt of two lines of salvage CIT in the control arm, ZUMA-7 trial (7) only allowed one line of salvage CIT. Sixth, only 66.4% of the patients in the BELINDA trial (8) had primary refractory disease as compared to 73.5% and 73.3% in ZUMA-7 (7) and TRANSFORM (6) trials, respectively. Our subgroup analysis based on limited data showed no potential effect modification by prior response status, i.e., between patients who had primary refractory disease and those who relapsed. (**eFigure 19 in the Supplementary**). Finally, the variable proportions of activated B-cell like (ABC) phenotype and germinal center B-cell (GCB) like lymphoma may have impacted the outcomes in these trials. Patients with ABC phenotype are known to harbor worse prognosis than those with GCB like lymphoma (14). Proportion of patients with ABC phenotype was greater in the tisa-cel arm compared to the SOC arm in the BELINDA trial (8). In contrast, approximately 23% and 32% had ABC phenotype in liso-cel and SOC arms, respectively in the TRANSFORM trial (6) and less than 10% of the patients exhibited the ABC phenotype in the ZUMA-7 trial (7). However, we did not find statistically significant effect modification by the cell of origin. A subgroup analysis of EFS by age group demonstrated similar efficacy among patients aged over 65 years (**eFigure 16 in the Supplementary**). These findings are supported by several encouraging reports on third-line CAR-T therapy among elderly patients with lymphoma, including pivotal studies and real-world data, which describe comparable outcomes for CAR-T therapy in older patients (15, 16).

Current evidence suggests a signal of potential OS benefit with CAR-T cell therapy compared to SOC. However, the difference was not statistically significant. Notably, a significant proportion of patients (52%) randomized to SOC arm were able to cross over to CAR-T cell therapy in the TRANSFORM (6) and BELINDA (8) trials or received commercial CAR-T cell therapy off study in the ZUMA-7 (7) trial. Similarly, no statistically significant difference was observed among axi-cel, liso-cel and tisa-cel though the direction of effect indicated potential superiority of axi-cel and liso-cel compared to tisa-cel. The lack of statistical significance in these comparisons could plausibly be explained by the fact that the OS analyses in the ZUMA-7 (7) and TRANSFORM (6) trials were interim as per protocol and hence, updated analyses based on mature OS data would provide more meaningful estimates. Toxicity profile of CAR-T cell therapy was consistent with the studies from third line setting. CAR-T cell therapy was not associated with an increased risk of all grade and grade ≥3 any AEs when compared to SOC, however, the type of adverse events were different in both arms (17, 18). The pooled incidences of grade ≥ 3 CRS and NT were 4.19% and 7.57% with CAR-T cell therapy which are consistent with the results of other studies. A detailed breakdown of adverse events observed with axi-cel, liso-cel, and tisa-cel is provided as **eTable 2 in the Supplementary**. Quality of life (QoL) is also an important endpoint to consider when opting CAR-T cell therapy. QoL report from the ZUMA-7 trial showed statistically significant improvement in QoL with axi-cel when compared to SOC at day 100. The results were consistent at day 150 (19). QoL data for CAR-T cell therapy in second line setting is still emerging and results from the TRANSFORM and BELINDA trials will inform further.

Moreover, a closer examination of the included trials revealed that majority of the patients were not able to proceed to ASCT in the SOC arm either because of suboptimal response to salvage chemotherapy or from progression of disease and only around 38.5% (166/431) of the patients underwent ASCT, whereas approximately 95.6% (415/434) of the patients, assigned to CAR-T cell therapy, were able to receive the CAR-T cell infusion (7, 8, 20). Acknowledging the limitations of our posthoc exploratory analysis, it is still an important finding that when directly compared, ASCT may be associated with an increased incidence of CR compared to CAR-T cell therapy. Consistent superiority of ASCT in patients who achieve at least a PR after salvage chemotherapy has been observed in a comparative analysis of Center for International Blood and Marrow Transplant Research (CIBMTR) registry by Shadman et al (21) which showed that ASCT was associated with a lower risk of relapse and an improved survival when compared to CAR-T cell therapy in R/R DLBCL patients. However, it should be noted that the analysis by Shadman et al (21) was retrospective in nature, and despite adequate adjustment for variable disease burden, different number of prior lines of therapy there could be potential confounding relationships. Also, it included patients who had exhibited chemosensitivity after salvage therapy compared to trials included in our analysis which included patients at the highest risk of chemo-refractory disease. Nevertheless, taken together these findings suggest that ASCT may still be preferrable for a subset of patients who exhibit sensitivity to salvage chemotherapy.

There are several noteworthy strengths of this study. First, we used a systematic approach to investigate the efficacy and safety of CAR-T cell therapy compared to SOC using totality of available evidence. Second, we performed a detailed and thorough review of relevant trials and provided a summary of baseline trial and population characteristics along with the limitations in each trial. Third, we conducted comprehensive pairwise analysis and used the GRADE approach to assess certainty of evidence for each patient important outcome and translated relative effects to absolute effect estimates. Lastly, we also conducted a network meta-analysis to assess comparative effectiveness of different CAR-T cell products using mixed treatment comparisons. However, this study is limited by a small number of included trials. Mixed treatment comparisons were based on an open network with sparse direct evidence which precluded the formal assessment of publication bias and incoherence. Median follow up durations varied across trials, and OS analyses in the ZUMA-7 (7) and TRANSFORM (6) trials were interim. Hence, mature OS data at longer follow up might offer different insights. We believe this study is timely as it examines data in totality, provides precise estimates for treatment effects of CAR-T cell therapy compared to SOC across different outcomes and presents a comprehensive assessment of comparative effectiveness of different CAR-T cell products.

## Conclusion

In summary, patients with R/R DLBCL harbor considerable disease heterogeneity and we need to tailor the choice of therapy carefully based on individual patient and associated disease factors. CAR-T cell therapy can be a potential second line treatment option for patients with primary refractory DLBCL or patients relapsing within 12 months of their first line chemoimmunotherapy. While patients relapsing more than 12 months after their first line treatment or those with chemosensitive disease may still benefit from ASCT.

## Data availability statement

The original contributions presented in the study are included in the article/**Supplementary Material**. Further inquiries can be directed to the corresponding authors.

## Author contributions

KA: Writing – original draft, Writing – review & editing. MZ: Writing – original draft, Writing – review & editing. EH: Writing – review & editing. AA: Writing – review & editing, Writing – original draft. SA: Writing – original draft, Writing – review & editing. NA: Writing – original draft, Writing – review & editing. AS: Writing – review & editing. JD: Writing – review & editing. JT: Writing – review & editing. BD: Writing – review & editing. AT: Writing – original draft, Writing – review & editing. MH: Writing – original draft, Writing – review & editing.
