# Supplementary material for: A systematic review and meta-analysis on utilizing anti-CD19 chimeric antigen receptor T-cell therapy as a second-line treatment for relapsed and refractory diffuse large B-cell lymphoma
Source: Front Oncol. 2024 Jul 18;14:1407001. doi: 10.3389/fonc.2024.1407001 (PMC11291309; doi:10.3389/fonc.2024.1407001)
Supplement: Supplementary file 1 [file DataSheet_1.pdf]

Online Supplementary Content

eMethods 1. PRISMA checklist

eMethods 2. Detailed search strategy

eTable 1. Outcome(s) definitions for the trials

eTable 2. Common adverse events, cytokine release syndrome events, and neurologic events

eFigure 1. PRISMA flowchart outlining the process of study selection

eFigure 2. Summary of risk of bias across included trials

eFigure 3. Forest plot showing adjusted event-free survival in CAR-T cell therapy vs. SOC

eFigure 4. Forest plot showing progression-free survival in CAR-T cell therapy vs. SOC

eFigure 5. Forest plot showing adjusted overall survival in CAR-T cell therapy vs. SOC

eFigure 6. Forest plot showing objective response rate in CAR-T cell therapy vs. SOC

eFigure 7. Forest plot showing complete response in CAR-T cell therapy vs. SOC

eFigure 8. Forest plot showing partial response in CAR-T cell therapy vs. SOC

eFigure 9. Analysis of complete response comparing patients who received CAR-T cell therapy with those who received standard of care and those who underwent autologous stem cell transplantation

eFigure 10. Forest plot showing all-grade adverse events in CAR-T cell therapy vs. SOC

eFigure 11. Forest plot showing grade ≥ 3 adverse events in CAR-T cell therapy vs. SOC

eFigure 12. Forest plot showing all grade cytokine release syndrome adverse events in patients receiving CAR-T cell therapy

eFigure 13. Forest plot showing grade ≥ 3-grade cytokine release syndrome adverse events in patients receiving CAR-T cell therapy

eFigure 14. Forest plot showing all grade neurotoxicity syndrome adverse events in patients receiving CAR-T cell therapy

eFigure 15. Forest plot showing grade ≥ 3 neurotoxicity syndrome adverse events in patients receiving CAR-T cell therapy

eFigure 16. Subgroup analysis by age for event-free survival outcome

eFigure 17. Subgroup analysis by non-Hodgkin lymphoma (NHL) subtypes for event-free survival

eFigure 18. Subgroup analysis by diffuse larger B-cell lymphoma molecular subtypes for event-free survival

eFigure 19. Subgroup analysis by the response to prior therapy for event-free survival

eFigure 20. Network plot

eFigure 21. Forest plot, league table, and p-score rankogram for mixed treatment comparisons for objective response rate.

eFigure 22. Forest plot, league table, and p-score rankogram for mixed treatment comparisons for complete response

eFigure 23. Forest plot, league table, and p-score rankogram for mixed treatment comparisons for grade ≥ 3 adverse events

eMethods 1. PRISMA checklist

Section and Topic	Item #	Checklist item	Location where item is reported
**TITLE**			
Title	1	Identify the report as a systematic review.	Page 1
**ABSTRACT**			
Abstract	2	See the PRISMA 2020 for Abstracts checklist.	Page 3
**INTRODUCTION**			
Rationale	3	Describe the rationale for the review in the context of existing knowledge.	Page 4
Objectives	4	Provide an explicit statement of the objective(s) or question(s) the review addresses.	Page 4
**METHODS**			
Eligibility criteria	5	Specify the inclusion and exclusion criteria for the review and how studies were grouped for the syntheses.	Page 5
Information sources	6	Specify all databases, registers, websites, organisations, reference lists and other sources searched or consulted to identify studies. Specify the date when each source was last searched or consulted.	Page 5. eMethods2 in supplement (page 6,7)
Search strategy	7	Present the full search strategies for all databases, registers and websites, including any filters and limits used.	Page 5, eMethods2 in the supplement (page 6,7)
Selection process	8	Specify the methods used to decide whether a study met the inclusion criteria of the review, including how many reviewers screened each record and each report retrieved, whether they worked independently, and if applicable, details of automation tools used in the process.	Page 5
Data collection process	9	Specify the methods used to collect data from reports, including how many reviewers collected data from each report, whether they worked independently, any processes for obtaining or confirming data from study investigators, and if applicable, details of automation tools used in the process.	Page 5
Data items	10a	List and define all outcomes for which data were sought. Specify whether all results that were compatible with each outcome domain in each study were sought (e.g., for all measures, time points, analyses), and if not, the methods used to decide which results to collect.	Page 6,7
	10b	List and define all other variables for which data were sought (e.g. participant and intervention characteristics, funding sources). Describe any assumptions made about any missing or unclear information.	Page 6,7
Study risk of bias assessment	11	Specify the methods used to assess risk of bias in the included studies, including details of the tool(s) used, how many reviewers assessed each study and whether they worked independently, and if applicable, details of automation tools used in the process.	Page 6
Effect measures	12	Specify for each outcome the effect measure(s) (e.g. risk ratio, mean difference) used in the synthesis or presentation of results.	Page 6,7
Synthesis methods	13a	Describe the processes used to decide which studies were eligible for each synthesis (e.g. tabulating the study intervention characteristics and comparing against the planned groups for each synthesis (item #5)).	Page 5
	13b	Describe any methods required to prepare the data for presentation or synthesis, such as handling of missing summary statistics, or data conversions.	Not applicable
	13c	Describe any methods used to tabulate or visually display results of individual studies and syntheses.	Page 6, 7
	13d	Describe any methods used to synthesize results and provide a rationale for the choice(s). If meta-analysis was performed, describe the model(s), method(s) to identify the presence and extent of statistical heterogeneity, and software package(s) used.	Page 6, 7

Section and Topic	Item #	Checklist item	Location where item is reported
	13e	Describe any methods used to explore possible causes of heterogeneity among study results (e.g. subgroup analysis, meta-regression).	Page 6,7
	13f	Describe any sensitivity analyses conducted to assess robustness of the synthesized results.	Page 6, 7
Reporting bias assessment	14	Describe any methods used to assess risk of bias due to missing results in a synthesis (arising from reporting biases).	Not applicable
Certainty assessment	15	Describe any methods used to assess certainty (or confidence) in the body of evidence for an outcome.	Page 7
**RESULTS**			
Study selection	16a	Describe the results of the search and selection process, from the number of records identified in the search to the number of studies included in the review, ideally using a flow diagram.	eFigure 1 in supplement (page 10)
	16b	Cite studies that might appear to meet the inclusion criteria, but which were excluded, and explain why they were excluded.	Not applicable
Study characteristics	17	Cite each included study and present its characteristics.	Table 1 page 8
Risk of bias in studies	18	Present assessments of risk of bias for each included study.	eFigure 2 in the supplement (page 11)
Results of individual studies	19	For all outcomes, present, for each study: (a) summary statistics for each group (where appropriate) and (b) an effect estimate and its precision (e.g. confidence/credible interval), ideally using structured tables or plots.	Page 8,9 eFigure 3-19 in the supplement (page 12-18)
Results of syntheses	20a	For each synthesis, briefly summarise the characteristics and risk of bias among contributing studies.	eFigure 2 in the supplement (page 11)
	20b	Present results of all statistical syntheses conducted. If meta-analysis was done, present for each the summary estimate and its precision (e.g. confidence/credible interval) and measures of statistical heterogeneity. If comparing groups, describe the direction of the effect.	Page 8-10 eFigure3-23 in the supplement(page 12-22)
	20c	Present results of all investigations of possible causes of heterogeneity among study results.	Page 9 eFigure 16 -19 in supplement (page 17,18)
	20d	Present results of all sensitivity analyses conducted to assess the robustness of the synthesized results.	Not applicable
Reporting biases	21	Present assessments of risk of bias due to missing results (arising from reporting biases) for each synthesis assessed.	eFigure 2 in the supplement (page 11)
Certainty of evidence	22	Present assessments of certainty (or confidence) in the body of evidence for each outcome assessed.	Table 2 page 11
**DISCUSSION**			
Discussion	23a	Provide a general interpretation of the results in the context of other evidence.	Page 11-15
	23b	Discuss any limitations of the evidence included in the review.	Page 15
	23c	Discuss any limitations of the review processes used.	Page 15
	23d	Discuss implications of the results for practice, policy, and future research.	Page 13,14
**OTHER INFORMATION**			
Registration	24a	Provide registration information for the review, including register name and registration	Not applicable

Section and Topic	Item #	Checklist item	Location where item is reported
and protocol		number, or state that the review was not registered.	
	24b	Indicate where the review protocol can be accessed, or state that a protocol was not prepared.	Not applicable
	24c	Describe and explain any amendments to information provided at registration or in the protocol.	Not applicable
Support	25	Describe sources of financial or non-financial support for the review, and the role of the funders or sponsors in the review.	Page 17
Competing interests	26	Declare any competing interests of review authors.	Page 17
Availability of data, code and other materials	27	Report which of the following are publicly available and where they can be found: template data collection forms; data extracted from included studies; data used for all analyses; analytic code; any other materials used in the review.	Not applicable

From: Page MJ, McKenzie JE, Bossuyt PM, Boutron I, Hoffmann TC, Mulrow CD, et al. The PRISMA 2020 statement: an updated guideline for reporting systematic reviews. BMJ 2021;372:n71. doi: 10.1136/bmj.n71

eMethods 2. Detailed search strategy

**Ovid MEDLINE(R) and Epub Ahead of Print, In-Process, In-Data-Review & Other Non-Indexed Citations and Daily <2017 to February 11, 2022>**		
**Embase <1974 to 2022 February 11>**		
**EBM Reviews - Cochrane Central Register of Controlled Trials <January 2022>**		
**EBM Reviews - Cochrane Database of Systematic Reviews <2005 to February 9, 2022>**		
1	Tisagenlecleucel.mp.	**2142**
2	Kymriah.mp.	**514**
3	Tisa-cel.mp.	**172**
4	Tis.mp.	**8647**
5	Axicabtagene ciloleucel.mp.	**1677**
6	Yescarta.mp.	**425**
7	KTE-C19.mp.	**105**
8	Axi-cel.mp.	**546**
9	Lisocabtagene maraleucel.mp.	**292**
10	Liso-cel.mp.	**132**
11	(receptors, antigen, t-cell or receptors, chimeric antigen or Antigens, CD19 or Tisagenlecleucel or Kymirah or Tisa-cel or Tis or Axicabtagene ciloleucel or Yescarta or KTE-C19 or Axi-cel or Lisocabtagene maraleucel).ti,ab,hw,kw.	**19214**
12	or/1-11	**19543**
13	("biologic response modifier therap*" or "biological response modifier therap*" or "chimeric immunoreceptors*" or "clonal antibod*" or "immune therap*" or "immunoglobulin therap*" or "immunological therap*" or "immunological treatment*" or "monoclonal antibod*" or "Antigen Receptors*" or "Chimeric Antigen *" or "Chimeric T-Cell Receptors*" or "Artificial T-Cell Receptors*" or "Chimeric Immunoreceptors*").ti.	**69658**
14	exp *Neoplasms/dt	**695294**
15	(cancer* or neoplasm* or neoplastic* or tumor* or tumor* or neoplasia* or carcinoma* or lymphoma* or burkitt* or relapsed* or refractory* or lymphoma* or large-cell* or Diffuse* or nonmelanoma* or metasta* or "Diffuse Large B Cell Lymphoma*" or "Diffuse Histiocytic Lymphomas*" or "Non-Hodgkin lymphoma*" or DLBCL* or LBCL*).ti.	**3720174**
16	14 or 15	**3953599**

17	13 and 16	**12279**
18	12 or 17	**31779**
19	Clinical Trial, Phase II/	**9495**
20	Clinical Trial, Phase III/	**7479**
21	phase 2 clinical trial.mp.	**166371**
22	phase 3 clinical trial.mp.	**141154**
23	Meta-Analysis/	**311534**
24	((meta adj analys*) or metaanalys* or (systematic* adj3 review*) or (("phase 2" or "phase II" or "phase 3" or "phase III") adj5 (trial or study))).ti,ab,kw.	**959557**
25	or/19-24	**1116874**
26	18 and 25	**2204**
27	((alpaca or alpacas or amphibian or amphibians or animal or animals or antelope or armadillo or armadillos or avian or baboon or baboons or beagle or beagles or bee or bees or bird or birds or bison or bovine or buffalo or buffaloes or buffalos or "c elegans" or "Caenorhabditis elegans" or camel or camels or canine or canines or carp or cats or cattle or chick or chicken or chickens or chicks or chimp or chimpanze or chimpanzees or chimps or cow or cows or "D melanogaster" or "dairy calf" or "dairy calves" or deer or dog or dogs or donkey or donkeys or drosophila or "Drosophila melanogaster" or duck or duckling or ducklings or ducks or equid or equids or equine or equines or feline or felines or ferret or ferrets or finch or finches or fish or flatworm or flatworms or fox or foxes or frog or frogs or "fruit flies" or "fruit fly" or "G mellonella" or "Galleria mellonella" or geese or gerbil or gerbils or goat or goats or goose or gorilla or gorillas or hamster or hamsters or hare or hares or heifer or heifers or horse or horses or insect or insects or jellyfish or kangaroo or kangaroos or kitten or kittens or lagomorph or lagomorphs or lamb or lambs or llama or llamas or macaque or macaques or macaw or macaws or marmoset or marmosets or mice or minipig or minipigs or mink or minks or monkey or monkeys or mouse or mule or mules or nematode or nematodes or octopus or octopuses or orangutan or "orang-utan" or orangutans or "orang-utans" or oxen or parrot or parrots or pig or pigeon or pigeons or piglet or piglets or pigs or porcine or primate or primates or quail or rabbit or rabbits or rat or rats or reptile or reptiles or rodent or rodents or ruminant or ruminants or salmon or sheep or shrimp or slug or slugs or swine or tamarin or tamarins or toad or toads or trout or urchin or urchins or vole or voles or waxworm or waxworms or worm or worms or xenopus or "zebra fish" or zebrafish) not (human or humans or patient or patients)).ti,ab,hw,kw.	**5863262**
28	26 not 27	**2202**
29	remove duplicates from 28	**1795**

eTable 1. Outcome(s) definitions for the trials

Outcome	ZUMA-7	TRANSFORM	BELINDA
EFS	Time from randomization to the earliest date of disease progression according to the Lugano classification, the commencement of new therapy for lymphoma, death from any cause, or a best response of stable disease up to and including the response on the day 150 assessment after randomization.	Time from randomization to death from any cause, progressive disease, failure to achieve complete or partial response by 9 weeks after randomization or start of new antineoplastic therapy due to efficacy concerns, whichever occurred first.	Time from randomization to stable or progressive disease at or after the week 12 assessment by the independent review committee according to the Lugano criteria or death at any time.
PFS	Time from randomization to disease progression or death from any cause.	Time from randomization to progressive disease or death from any cause, whichever occurs first.	NR

Abbreviations: EFS: event-free survival; PFS: progression-free survival; NR: not reported

eTable 2. Common adverse events, cytokine release syndrome events, and neurologic events

	ZUMA-7				TRANSFORM				BELINDA			
	Axi-cel N=170		Standard of Care N=168		Liso-cel N=92		Standard of Care N=91		Tisagenlecleucel N=162		Standard of Care N=160	
**Event[a]**	Any Grade no. (%)	Grade ≥3 no. (%)	Any Grade no. (%)	Grade ≥3 no. (%)	Grade 1-2[b] no. (%)	Grade ≥3 no. (%)	Grade 1-2[b] no. (%)	Grade ≥3 no. (%)	Any Grade no. (%)	Grade ≥3 no. (%)	Any Grade no. (%)	Grade ≥3 no. (%)
Any adverse event[c]	170 (100)	155 (91)	168 (100)	140 (83)	90 (98)	85 (92)	90 (99)	79 (87)	160(98.8)	136 (84)	158(98.8)	144 (90)
Abdominal pain	NA	NA	NA	NA	11 (12)	3 (3)	11 (12)	1 (1)	17 (10.5)	2 (1.2)	30 (18.8)	5 (3.1)
Anemia	71 (42)	51 (30)	91 (54)	65 (39)	36 (39)	45 (49)	34 (37)	45 (49)	80 (49.4)	54(33.3)	115(71.9)	92(57.5)
Arthralgia	NA	NA	NA	NA	13 (14)	0	9 (10)	0	NA	NA	NA	NA
Asthenia	NA	NA	NA	NA	9 (10)	1 (1)	8 (9)	0	8 (4.9)	1 (0.6)	19 (11.9)	2 (1.3)
Back pain	NA	NA	NA	NA	14 (15)	1 (1)	15 (16)	2 (2)	NA	NA	NA	NA
Blood creatinine increase	NA	NA	NA	NA	NA	NA	NA	NA	29 (17.9)	2 (1.2)	29 (18.1)	1 (0.6)
Bone pain	NA	NA	NA	NA	12 (13)	0	9 (10)	0	NA	NA	NA	NA
Chills	47(28)	1 (1)	14 (8)	0	NA	NA	NA	NA	NA	NA	NA	NA
Constipation	34 (20)	0	58 (35)	0	31 (34)	2 (2)	22 (24)	0	48 (29.6)	0	42 (26.3)	0
Cough	42 (25)	1 (1)	18 (11)	0	13 (14)	0	8 (9)	0	18 (11.1)	0	13 (8.1)	0
Decreased appetite	42 (25)	7 (4)	42 (25)	6 (4)	21 (23)	1 (1)	26 (29)	3 (3)	21 (13.0)	1 (0.6)	17 (10.6)	1 (0.6)
Diarrhea	71 (42)	4 (2)	66 (39)	7 (4)	23 (25)	0	37 (41)	3 (3)	35 (21.6)	3 (1.9)	58 (36.3)	6 (3.8)
Dizziness	36 (21)	2 (1)	21 (12)	1 (1)	20 (22)	0	13 (14)	0	19 (11.7)	0	14 (8.8)	0
Dyspepsia	NA	NA	NA	NA	5 (5)	0	10 (11)	0	NA	NA	NA	NA
Dyspnea	NA	NA	NA	NA	12 (13)	1 (1)	7 (8)	1 (1)	15 (9.3)	2 (1.2)	21 (13.1)	2 (1.3)
Edema peripheral	NA	NA	NA	NA	15 (16)	1 (1)	16 (18)	0	18 (11.1)	0	18 (11.3)	0
Fatigue	71 (42)	11 (6)	87 (52)	4 (2)	36 (39)	0	34 (37)	2 (2)	38 (23.5)	3 (1.9)	49 (30.6)	6 (3.8)
Febrile neutropenia	4 (2)	4 (2)	46 (27)	46 (27)	4 (4)	11 (12)	3 (3)	19 (21)	21 (13.0)	21 (13.0)	40 (25.0)	40 (25.0)
Headache	70 (41)	5 (3)	43 (26)	2 (1)	39 (42)	4 (4)	19 (21)	1 (1)	37 (22.8)	0	32 (20.0)	1 (0.6)
Hypertension	NA	NA	NA	NA	9 (10)	4 (4)	6 (7)	1 (1)	NA	NA	NA	NA
Hypokalemia	44 (26)	10 (6)	49 (29)	11 (7)	16 (17)	4 (4)	19 (21)	4 (4)	45 (27.8)	8 (4.9)	49 (30.6)	14 (8.8)
Hypomagnesemia	NA	NA	NA	NA	13 (14)	0	19 (21)	1 (1)	20 (12.3)	0	29 (18.1)	3 (1.9)
Hypophosphatemia	45 (26)	31 (18)	29 (17)	21 (12)	6 (7)	3 (3)	10 (11)	6 (7)	13 (8.0)	6 (3.7)	17 (10.6)	4 (2.5)
Hypotension	75 (44)	19 (11)	25 (15)	5 (3)	18 (20)	3 (3)	4 (4)	0	NA	NA	NA	NA
Hypoxia	37 (22)	16 (9)	13 (8)	7 (4)	NA	NA	NA	NA	NA	NA	NA	NA
Insomnia	NA	NA	NA	NA	19 (21)	0	11 (12)	0	NA	NA	NA	NA
Leukopenia	55 (32)	50 (29)	43 (26)	37 (22)	9 (10)	14 (15)	6 (7)	11 (12)	22 (13.6)	21(13.0)	30 (18.8)	28(17.5)
Lymphopenia	NA	NA	NA	NA	8 (9)	23 (25)	4 (4)	8 (9)	NA	NA	NA	NA
Mucosal inflammation	NA	NA	NA	NA	4 (4)	0	11 (12)	3 (3)	5 (3.1)	1 (0.6)	18 (11.3)	0
Myalgia	NA	NA	NA	NA	11 (12)	1 (1)	4 (4)	0	NA	NA	NA	NA
Nausea	69 (41)	3 (2)	116 (69)	9 (5)	49 (53)	3 (3)	52 (57)	3 (3)	67 (41.4)	2 (1.2)	79 (49.4)	10 (6.3)
Neutropenia	121 (71)	118 (69)	70 (42)	69 (41)	43 (47)	74 (80)	17 (19)	46 (51)	67 (41.4)	65(40.1)	65 (40.6)	63(39.4)
Peripheral sensory neuropathy	NA	NA	NA	NA	7 (8)	0	11 (12)	0	NA	NA	NA	NA
Prolonged cytopenia	NA	NA	NA	NA	NA	40 (43)	NA	3 (3)	NA	NA	NA	NA
Pyrexia	158 (93)	15 (9)	43 (26)	1 (1)	27 (29)	0	21 (23)	0	42 (25.9)	0	50 (31.3)	3 (1.9)
Sinus tachycardia	58 (34)	3 (2)	17 (10)	1 (1)	9 (10)	0	10 (11)	0	NA	NA	NA	NA
Stomatitis	NA	NA	NA	NA	5 (5)	0	9 (10)	2 (2)	NA	NA	NA	NA
Thrombocytopenia	50 (29)	25 (15)	101 (60)	95 (57)	30 (33)	45 (49)	35 (38)	58 (64)	59 (36.4)	52(32.1)	79 (49.4)	76(47.5)
Tremor	NA	NA	NA	NA	11 (12)	1 (1)	0	0	NA	NA	NA	NA
Vomiting	33 (19)	0	55 (33)	1 (1)	18 (20)	1 (1)	21 (23)	2 (2)	24 (14.8)	1 (0.6)	35 (21.9)	3 (1.9)
**Cytokine release syndrome[d]**	157 (92)	11 (6)	NA	NA	45 (49)	1 (1)	0	0	95 (58.6)	8 (4.9)	0	0
Chills	38 (24)	0	NA	NA	NA	NA	NA	NA	NA	NA	NA	NA
Headache	32 (20)	2 (1)	NA	NA	NA	NA	NA	NA	NA	NA	NA	NA
Hypotension	68 (43)	18 (11)	NA	NA	NA	NA	NA	NA	NA	NA	NA	NA
Hypoxia	31 (20)	13 (8)	NA	NA	NA	NA	NA	NA	NA	NA	NA	NA
Pyrexia	155 (99)	14 (9)	NA	NA	NA	NA	NA	NA	NA	NA	NA	NA
Sinus tachycardia	49 (31)	3 (2)	NA	NA	NA	NA	NA	NA	NA	NA	NA	NA
**Neurologic event[e]**	102 (60)	36 (21)	33(20)	1(1)	11 (12)	4 (4)	NA	NA	16 (10.3)	3 (1.9)	NA	NA
Aphasia	36 (21)	12 (7)	0	0	NA	NA	NA	NA	NA	NA	NA	NA
Confusional state	40 (24)	9 (5)	4 (2)	0	NA	NA	NA	NA	NA	NA	NA	NA
Delirium	3 (2)	3 (2)	5 (3)	1 (1)	NA	NA	NA	NA	NA	NA	NA	NA
Encephalopathy	29 (17)	20 (12)	2 (1)	0	NA	NA	NA	NA	NA	NA	NA	NA
Paresthesia	8 (5)	1 (1)	14 (8)	0	NA	NA	NA	NA	NA	NA	NA	NA
Tremor	44 (26)	2 (1)	1 (1)	0	NA	NA	NA	NA	NA	NA	NA	NA

[a] Cell count decrease was not included

[b] Grade 1-2 reported for TRANSFORM trial

[c] For BELINDA trial, this corresponds to 'Number of patients with at least 1 event'

[d] The percentage of each cytokine release syndrome (CRS) event was relative to the total CRS observed i.e., 157 for ZUMA-7 trial for all grades

[e] BELINDA trial reported the proportion of neurological events in Tisagenlecleucel arm relative to 155 patients (reported here). However, the total number of participants were 162

Abbreviations: Axi-cel, Axicabtagene ciloleucel; Liso-cel, Lisocabtagene maraleucel; N, number of participants; NA, not applicable

eFigure 1. PRISMA flowchart outlining the process of study selection

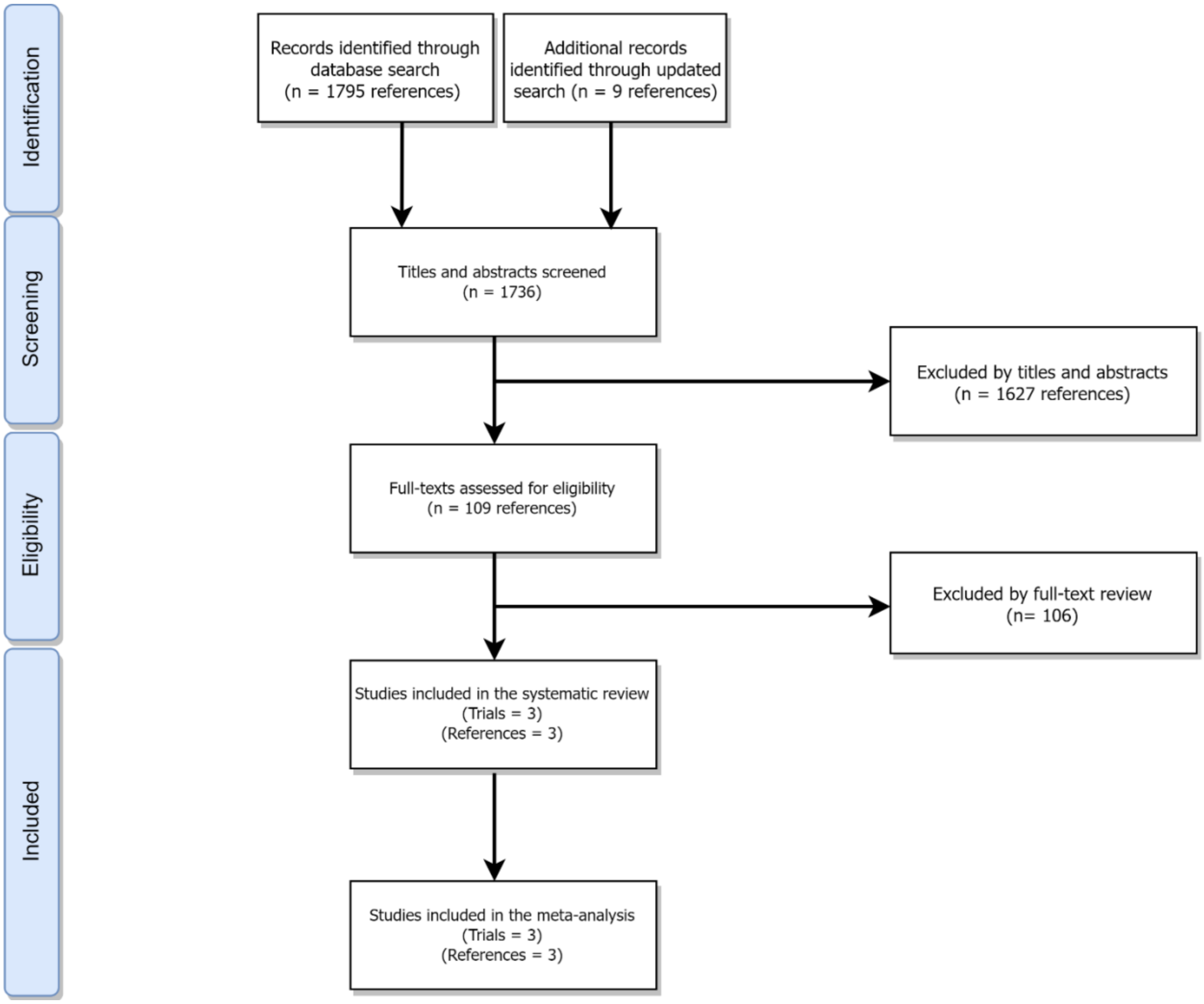

eFigure 2. Summary of risk of bias across included trials

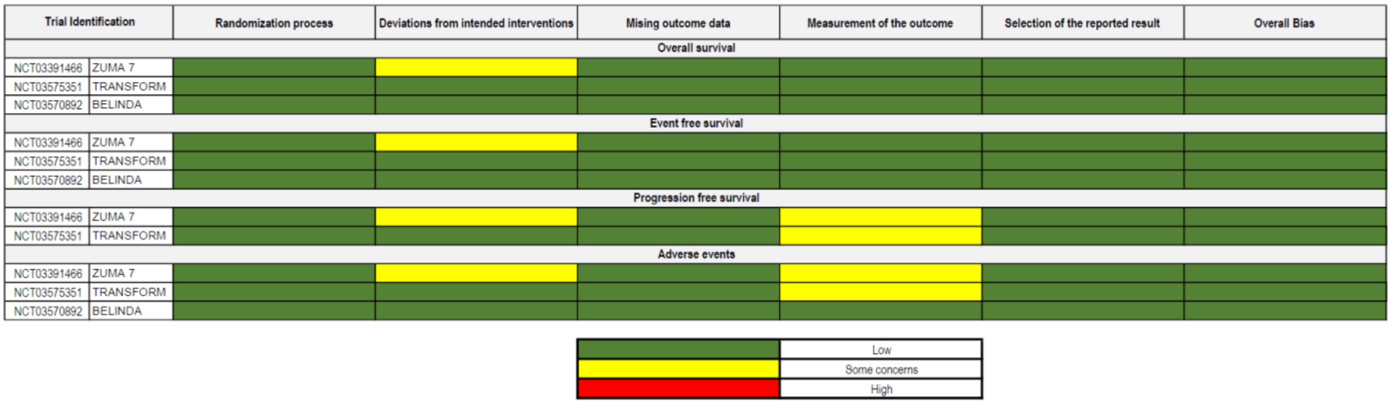

eFigure 3. Forest plot showing adjusted event-free survival in CAR-T cell therapy vs. SOC

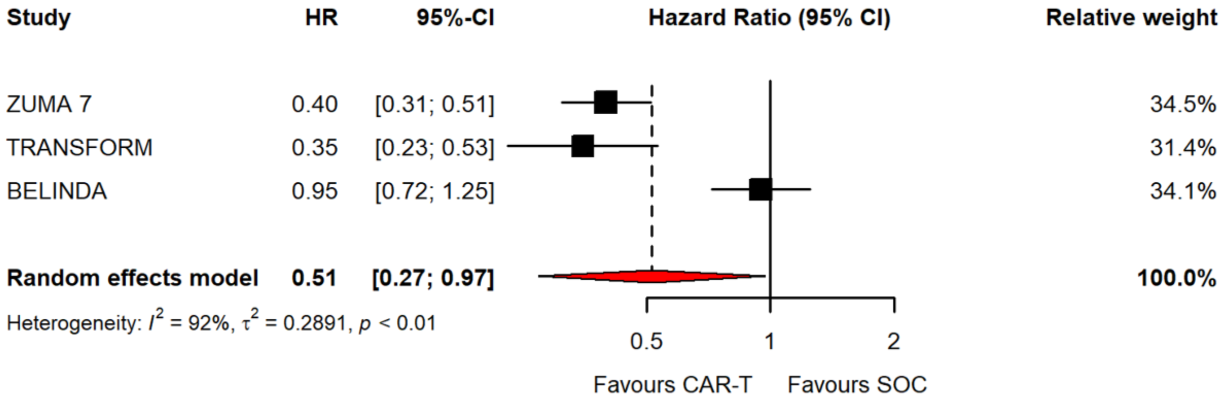

eFigure 4. Forest plot showing progression-free survival in CAR-T cell therapy vs. SOC

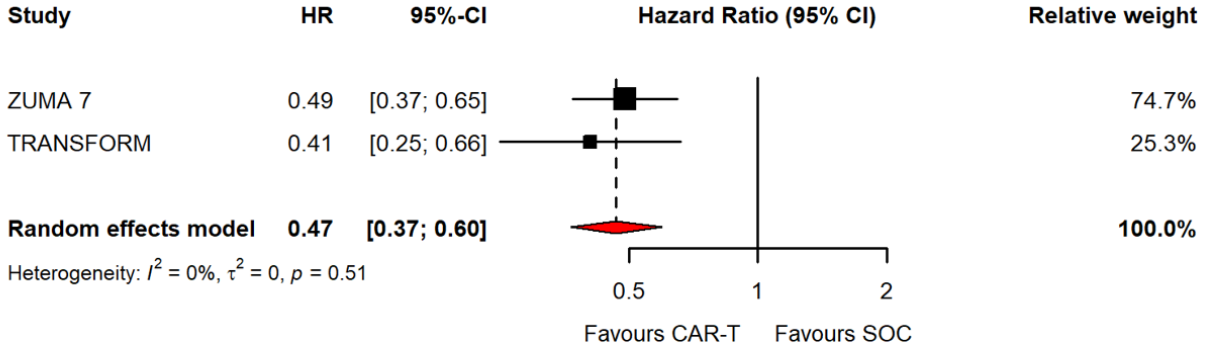

eFigure 5. Forest plot showing adjusted overall survival in CAR-T cell therapy vs. SOC

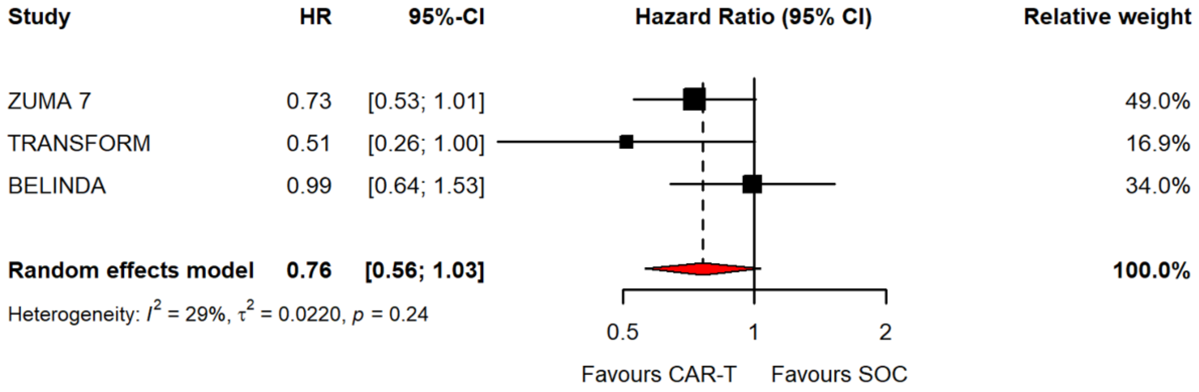

Abbreviations: CAR-T: chimeric antigenic receptor T-cell therapy; SOC: standard of care

eFigure 6. Forest plot showing objective response rate in CAR-T cell therapy vs. SOC

Study	CAR-T Events	CAR-T Total	SOC Events	SOC Total	RR	95%-CI	Relative weight
ZUMA 7	150	180	90	179	1.66	[1.41; 1.94]	36.4%
TRANSFORM	79	92	44	92	1.80	[1.43; 2.26]	32.3%
BELINDA	75	162	68	160	1.09	[0.85; 1.39]	31.3%
**Random effects model**	**304**	**434**	**202**	**431**	**1.49**	**[1.13; 1.97]**	**100.0%**

Heterogeneity: $I^2 = 81\%$, $\tau^2 = 0.0481$, $p < 0.01$

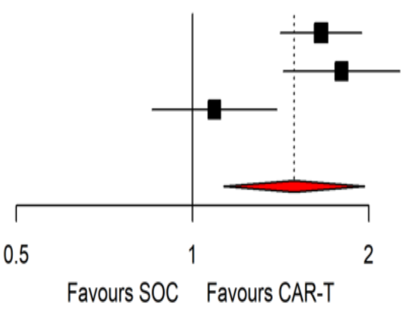

eFigure 7. Forest plot showing complete response in CAR-T cell therapy vs. SOC

Study	CAR-T Events	CAR-T Total	SOC Events	SOC Total	RR	95%-CI	Relative weight
ZUMA 7	117	180	58	179	2.01	[1.58; 2.54]	36.0%
TRANSFORM	61	92	36	92	1.69	[1.26; 2.27]	33.4%
BELINDA	46	162	44	160	1.03	[0.73; 1.47]	30.6%
**Random effects model**	**224**	**434**	**138**	**431**	**1.55**	**[1.07; 2.24]**	**100.0%**

Heterogeneity: $I^2 = 79\%$, $\tau^2 = 0.0830$, $p < 0.01$

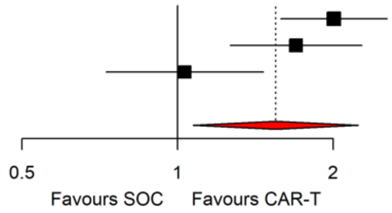

eFigure 8. Forest plot showing partial response in CAR-T cell therapy vs. SOC

Study	CAR-T Events	CAR-T Total	SOC Events	SOC Total	RR	95%-CI	Relative weight
ZUMA 7	33	180	32	179	1.03	[0.66; 1.59]	43.1%
TRANSFORM	18	92	8	92	2.25	[1.03; 4.91]	19.4%
BELINDA	29	162	24	160	1.19	[0.73; 1.96]	37.5%
**Random effects model**	**80**	**434**	**64**	**431**	**1.26**	**[0.86; 1.85]**	**100.0%**

Heterogeneity: $I^2 = 33\%$, $\tau^2 = 0.0381$, $p = 0.23$

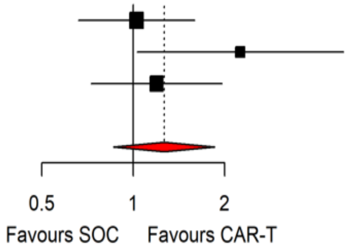

Abbreviations: CAR-T: chimeric antigenic receptor T-cell therapy; SOC: standard of care

eFigure 9. Analysis of complete response comparing patients who received CAR-T cell therapy with those who received standard of care and those who underwent autologous stem cell transplantation

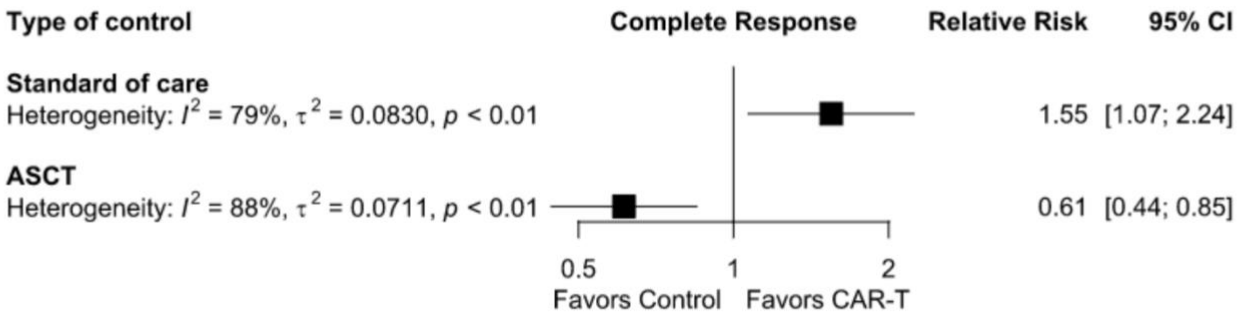

Type of control	№ of participants (studies)	Relative effect (95% CI)	Anticipated absolute effects* (95% CI)	
			CR with Control	Risk difference with CAR-T
Standard of care (SOC)	865 (3 RCTs)	**RR 1.55** (1.07 to 2.24)	320 per 1,000	**176 more per 1,000** (from 22 more to 397 more)
Autologous stem cell transplant (ASCT)	582 (3 RCTs)	**RR 0.61** (0.44 to 0.85)	831 per 1,000	**324 fewer per 1,000** (from 466 more to 125 more)

Abbreviations: CAR-T: chimeric antigenic receptor T-cell therapy; CR: complete response

eFigure 10. Forest plot showing all adverse events in CAR-T cell therapy vs. SOC

Study	CAR-T Events	CAR-T Total	SOC Events	SOC Total	RR	95%-CI	Relative weight
ZUMA 7	170	170	168	168	1.00	[0.99; 1.01]	42.2%
TRANSFORM	92	92	85	91	1.07	[1.01; 1.13]	21.4%
BELINDA	160	162	158	160	1.00	[0.98; 1.02]	36.4%
**Random effects model**	**422**	**424**	**411**	**419**	**1.01**	**[0.98; 1.05]**	**100.0%**

Heterogeneity: I^2 = 82%, τ^2 = 0.0007, p < 0.01

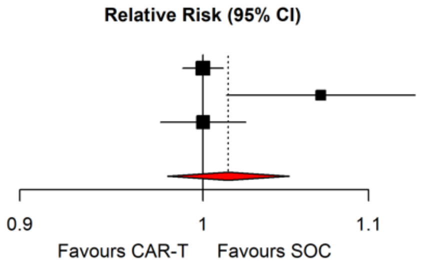

eFigure 11. Forest plot showing grade ≥ 3 adverse events in CAR-T cell therapy vs. SOC

Study	CAR-T Events	CAR-T Total	SOC Events	SOC Total	RR	95%-CI	Relative weight
ZUMA 7	155	170	140	168	1.09	[1.01; 1.19]	33.6%
TRANSFORM	90	92	79	91	1.13	[1.03; 1.23]	33.1%
BELINDA	136	162	144	160	0.93	[0.86; 1.02]	33.2%
**Random effects model**	**381**	**424**	**363**	**419**	**1.05**	**[0.93; 1.18]**	**100.0%**

Heterogeneity: I^2 = 82%, τ^2 = 0.0085, p < 0.01

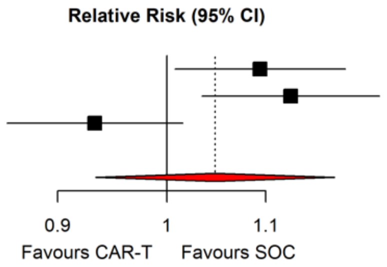

eFigure 12. Forest plot showing all grade cytokine release syndrome adverse events in patients receiving CAR-T cell therapy

Study	Events	Total	Events	95%-CI	Relative weight
ZUMA 7	157	170	92.35	[87.28; 95.87]	33.5%
TRANSFORM	45	92	48.91	[38.34; 59.56]	33.0%
BELINDA	95	155	61.29	[53.14; 69.00]	33.5%
**Random effects model**	**297**	**417**	**69.83**	**[39.53; 92.88]**	**100.0%**

Heterogeneity: I^2 = 97%, τ^2 = 0.0716, p < 0.01

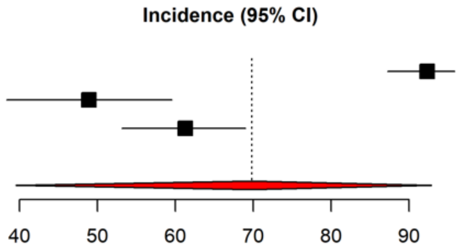

Abbreviations: CAR-T: chimeric antigenic receptor T-cell therapy; SOC: standard of care

eFigure 13. Forest plot showing grade ≥ 3 cytokine release syndrome adverse events in patients receiving CAR-T cell therapy

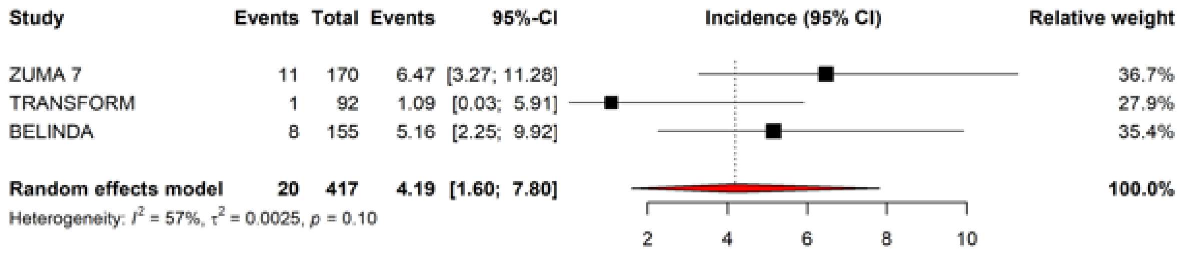

eFigure 14. Forest plot showing all grade neurotoxicity syndrome adverse events in patients receiving CAR-T cell therapy

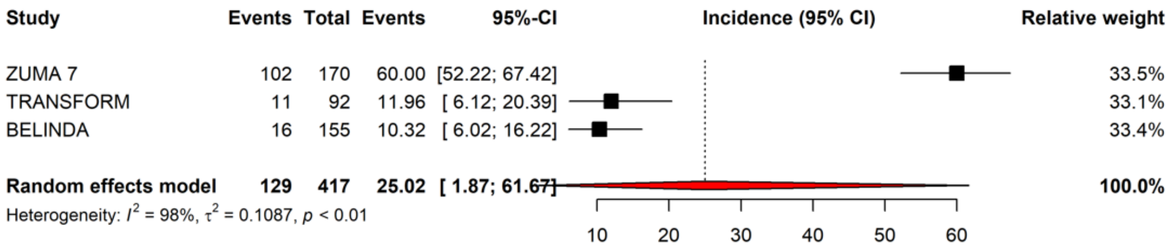

eFigure 15. Forest plot showing grade ≥ 3 neurotoxicity syndrome adverse events in patients receiving CAR-T cell therapy

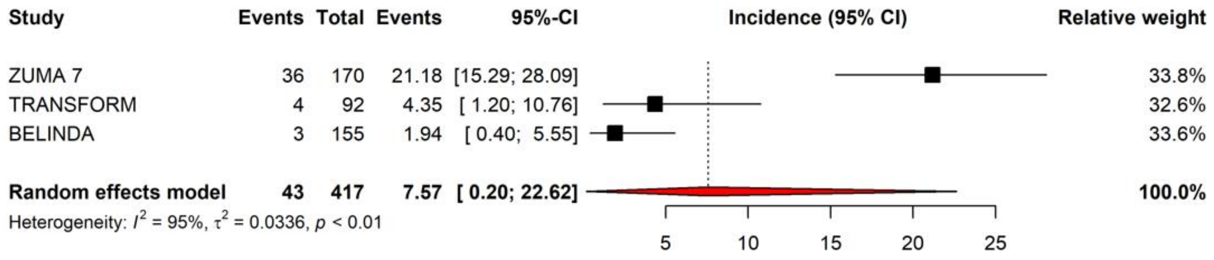

Abbreviations: CAR-T: chimeric antigenic receptor T-cell therapy

eFigure 16. Subgroup analysis by age for event-free survival outcome

Study	HR	95%-CI	Relative weight
**subgroup = <65yr**			
ZUMA-7	0.49	[0.36; 0.67]	39.6%
TRANSFORM	0.28	[0.16; 0.49]	22.9%
**Random effects model**	**0.39**	**[0.23; 0.67]**	**62.5%**
Heterogeneity: I^2 = 66%, τ^2 = 0.1033, p = 0.09			
**subgroup = >65yr**			
ZUMA-7	0.28	[0.17; 0.47]	24.5%
TRANSFORM	0.30	[0.13; 0.70]	12.9%
**Random effects model**	**0.29**	**[0.18; 0.45]**	**37.5%**
Heterogeneity: I^2 = 0%, τ^2 = 0, p = 0.89			
**Random effects model**	**0.35**	**[0.25; 0.50]**	**100.0%**
Heterogeneity: I^2 = 43%, τ^2 = 0.0523, p = 0.15			
Test for subgroup differences: χ^2_1 = 0.76, df = 1 (p = 0.38)			

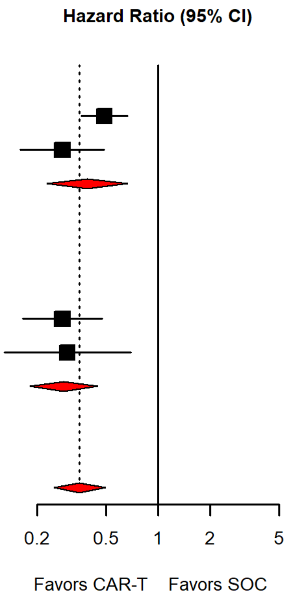

eFigure 17. Subgroup analysis by non-Hodgkin lymphoma (NHL) subtypes for event-free survival

Study	HR	95%-CI	Relative weight
**subgroup = DLBCL**			
ZUMA-7	0.44	[0.32; 0.60]	60.4%
TRANSFORM	0.36	[0.20; 0.64]	18.1%
**Random effects model**	**0.42**	**[0.32; 0.55]**	**78.6%**
Heterogeneity: I^2 = 0%, τ^2 = 0, p = 0.55			
**subgroup = HGBCL**			
ZUMA-7	0.28	[0.14; 0.57]	11.5%
TRANSFORM	0.41	[0.19; 0.89]	9.9%
**Random effects model**	**0.33**	**[0.20; 0.57]**	**21.4%**
Heterogeneity: I^2 = 0%, τ^2 = 0, p = 0.48			
**Random effects model**	**0.40**	**[0.31; 0.51]**	**100.0%**
Heterogeneity: I^2 = 0%, τ^2 = 0, p = 0.70			
Test for subgroup differences: χ^2_1 = 0.57, df = 1 (p = 0.45)			

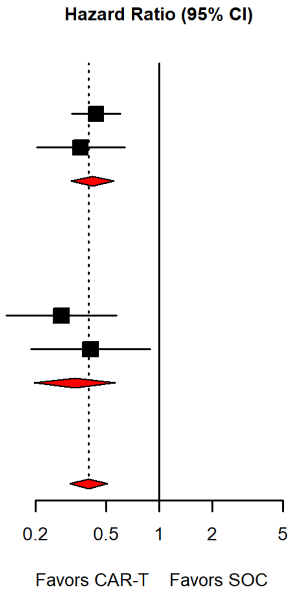

Abbreviations: CAR-T: chimeric antigenic receptor T-cell therapy; SOC: standard of care

eFigure 18. Subgroup analysis by diffuse larger B-cell lymphoma molecular subtypes for event-free survival

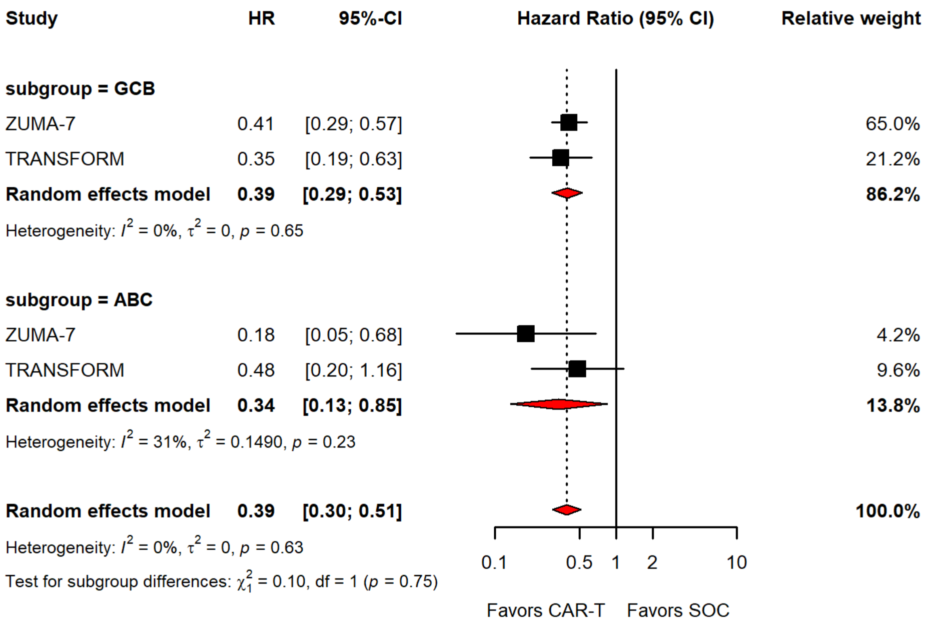

eFigure 19. Subgroup analysis by the response to prior therapy for event-free survival

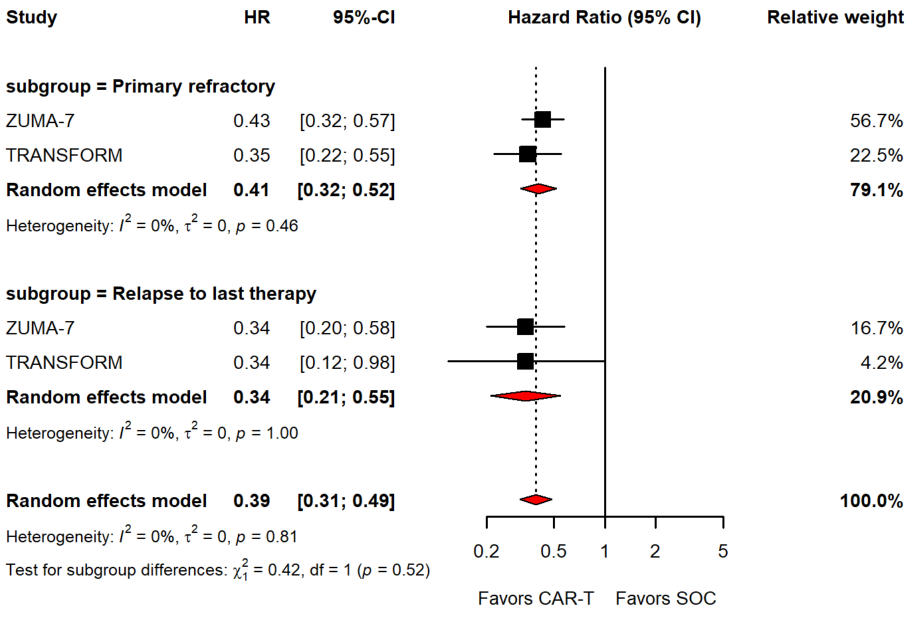

Abbreviations: CAR-T: chimeric antigenic receptor T-cell therapy; SOC: standard of care

eFigure 20. Network plot

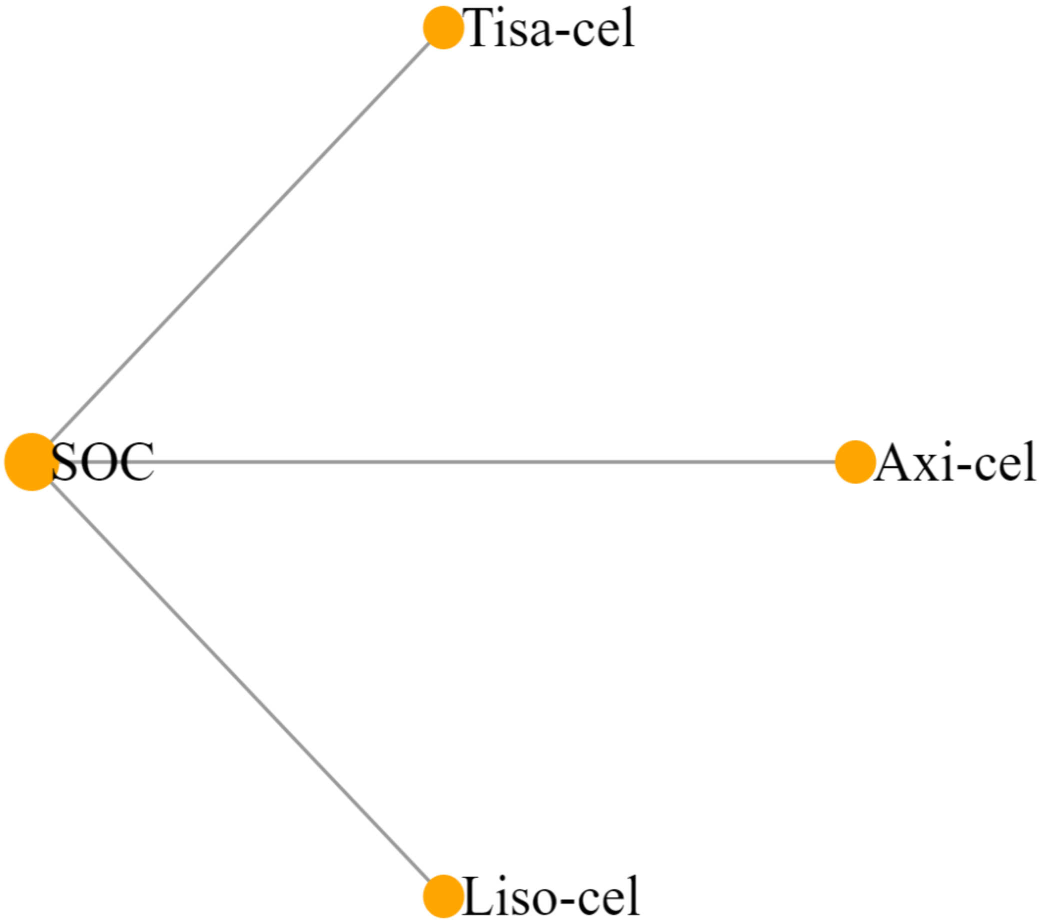

Abbreviations: Axi-cel: Axicabtagene ciloleucel; Tisa-cel: Tisagenlecleucel; Liso-cel: Lisocabtagene maraleucel; SOC: Standard of care

eFigure 21. Forest plot, league table, and p-score rankogram for mixed treatment comparisons for objective response rate.

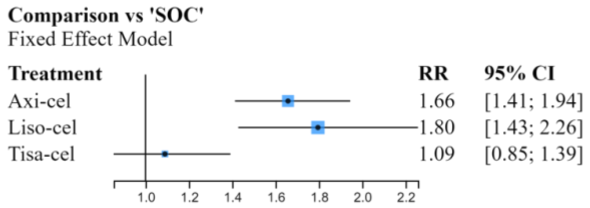

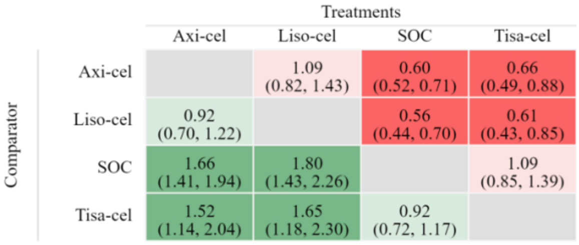

Comparator \ Treatments	Axi-cel	Liso-cel	SOC	Tisa-cel
Axi-cel		1.09 (0.82, 1.43)	0.60 (0.52, 0.71)	0.66 (0.49, 0.88)
Liso-cel	0.92 (0.70, 1.22)		0.56 (0.44, 0.70)	0.61 (0.43, 0.85)
SOC	1.66 (1.41, 1.94)	1.80 (1.43, 2.26)		1.09 (0.85, 1.39)
Tisa-cel	1.52 (1.14, 2.04)	1.65 (1.18, 2.30)	0.92 (0.72, 1.17)	

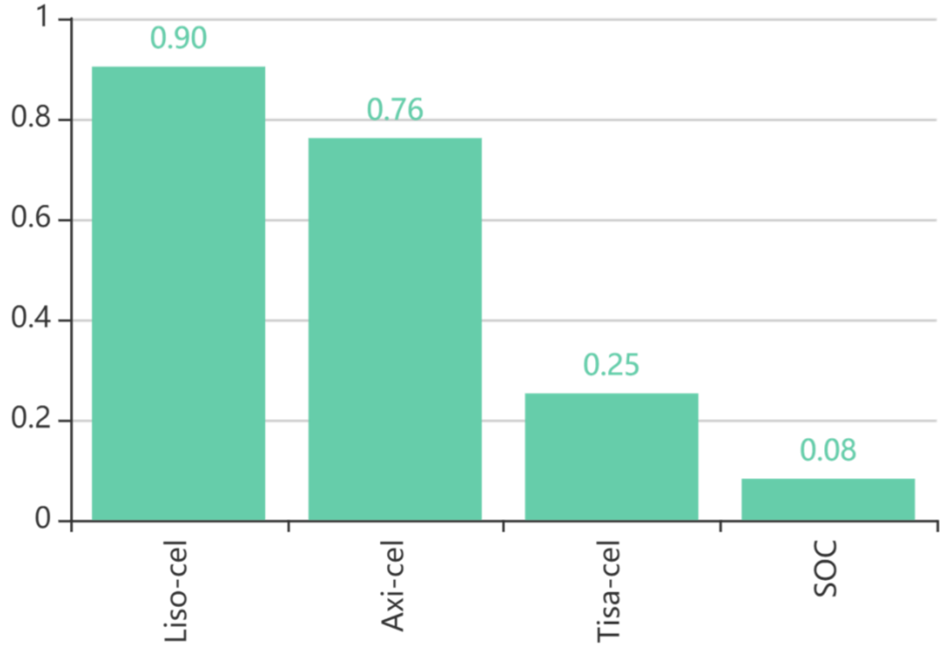

Abbreviations: Axi-cel: Axicabtagene ciloleucel; Tisa-cel: Tisagenlecleucel; Liso-cel: Lisocabtagene maraleucel; SOC: Standard of care

eFigure22. Forest plot, league table, and p-score rankogram for mixed treatment comparisons for complete response

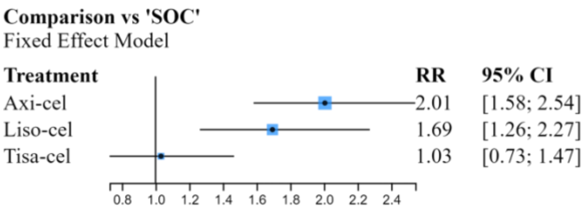

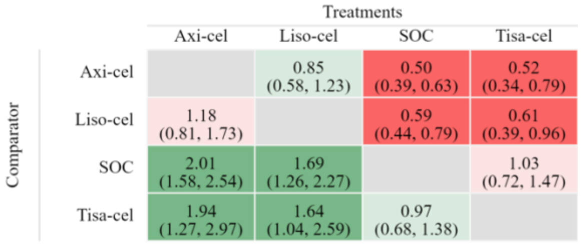

Comparator	Axi-cel	Liso-cel	SOC	Tisa-cel
Axi-cel		0.85 (0.58, 1.23)	0.50 (0.39, 0.63)	0.52 (0.34, 0.79)
Liso-cel	1.18 (0.81, 1.73)		0.59 (0.44, 0.79)	0.61 (0.39, 0.96)
SOC	2.01 (1.58, 2.54)	1.69 (1.26, 2.27)		1.03 (0.72, 1.47)
Tisa-cel	1.94 (1.27, 2.97)	1.64 (1.04, 2.59)	0.97 (0.68, 1.38)	

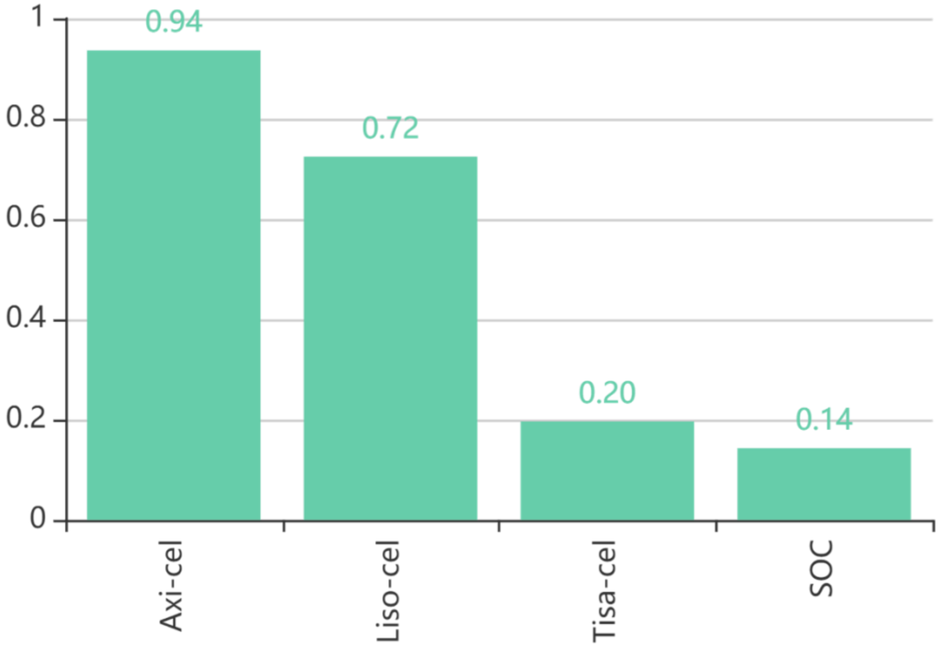

Abbreviations: Axi-cel: Axicabtagene ciloleucel; Tisa-cel: Tisagenlecleucel; Liso-cel: Lisocabtagene maraleucel; SOC: Standard of care

eFigure23. Forest plot, league table, and p-score rankogram for mixed treatment comparisons for grade ≥ 3 adverse events

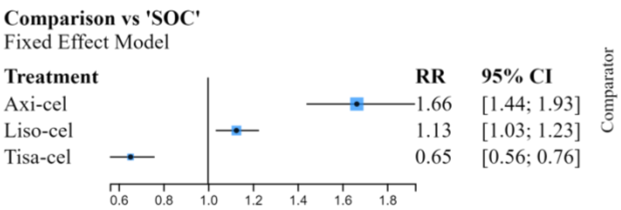

Comparator \ Treatments	Axi-cel	Liso-cel	SOC	Tisa-cel
Axi-cel		0.68 (0.57, 0.80)	0.60 (0.52, 0.69)	0.39 (0.32, 0.49)
Liso-cel	1.48 (1.25, 1.75)		0.88 (0.81, 0.97)	0.58 (0.49, 0.69)
SOC	1.66 (1.44, 1.93)	1.13 (1.03, 1.23)		0.65 (0.56, 0.76)
Tisa-cel	2.55 (2.06, 3.14)	1.72 (1.45, 2.05)	1.53 (1.31, 1.78)	

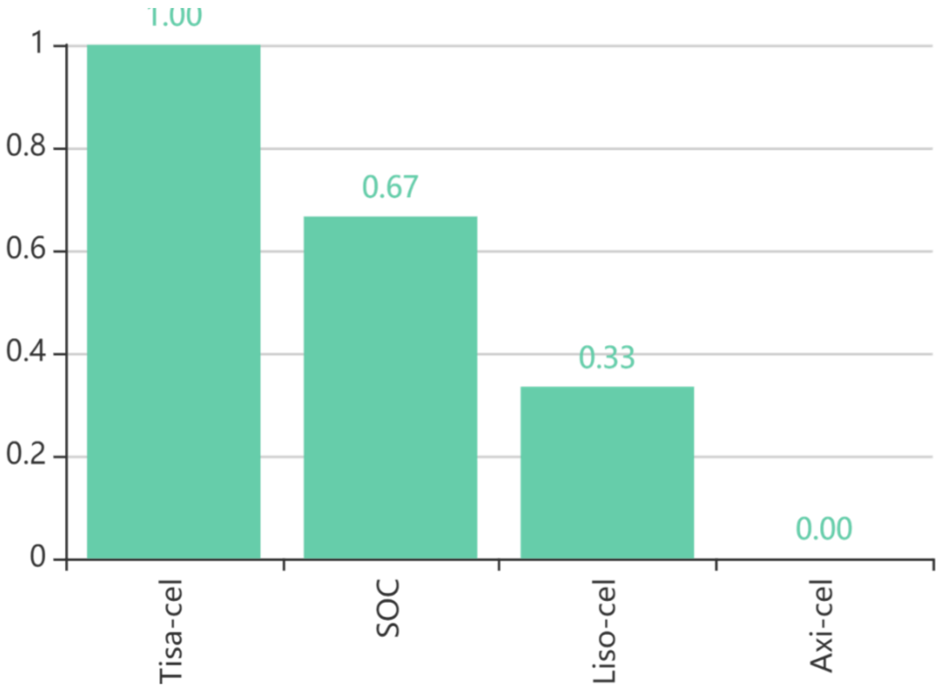

Abbreviations: Axi-cel: Axicabtagene ciloleucel; Tisa-cel: Tisagenlecleucel; Liso-cel: Lisocabtagene maraleucel; SOC: Standard of care
